# The *Toxoplasma* Effector GRA4 Hijacks Host TBK1 to Oppositely Regulate Anti‐*T. Gondii* Immunity and Tumor Immunotherapy

**DOI:** 10.1002/advs.202400952

**Published:** 2024-06-21

**Authors:** Zhiqiang Hu, Yufen Zhang, Yingchao Xie, Jianwu Yang, Haotian Tang, Bolin Fan, Ke Zeng, Zhongxin Han, Jiansen Lu, Huaji Jiang, Wenqiang Peng, Hongyu Li, Huadan Chen, Sha Wu, Bang Shen, Zhao‐Rong Lun, Xiao Yu

**Affiliations:** ^1^ Department of Immunology, School of Basic Medical Sciences Southern Medical University Guangzhou 510515 China; ^2^ Zhejiang Provincial Key Laboratory of Pancreatic Disease, The First Affiliated Hospital, Institute of Translational Medicine Zhejiang University School of Medicine Zhejiang University Hangzhou 310029 China; ^3^ State Key Laboratory of Biocontrol, School of Life Sciences Sun Yat‐sen University Guangzhou 510275 China; ^4^ State Key Laboratory of Agricultural Microbiology, College of Veterinary Medicine Huazhong Agricultural University Wuhan 430070 China; ^5^ Department of Joint Surgery the Fifth Affiliated Hospital of Southern Medical University Guangzhou 510900 China; ^6^ Yue Bei People's Hospital Postdoctoral Innovation Practice Base Southern Medical University Guangzhou 510515 China; ^7^ Guangdong Provincial Key Laboratory of Proteomics Southern Medical University Guangzhou 510515 China; ^8^ Department of Clinical Laboratory Medicine, Guangdong Provincial People’s Hospital (Guangdong Academy of Medical Sciences) Southern Medical University Guangzhou 510000 China

**Keywords:** attenuated T. gondii, selective autophagy, toxoplasmosis, tumor therapy, type I interferon

## Abstract

*Toxoplasma gondii* (*T. gondii*)‐associated polymorphic effector proteins are crucial in parasite development and regulating host anti‐*T. gondii* immune responses. However, the mechanism remains obscure. Here, it is shown that *Toxoplasma* effector dense granules 4 (GRA4) restricts host IFN‐I activation. Infection with Δ*gra4* mutant *T. gondii* strain induces stronger IFN‐I responses and poses a severe threat to host health. Mechanistically, GRA4 binds to phosphorylated TBK1 to promote TRIM27‐catalyzed K48‐ubiquitination at Lys251/Lys372 residues, which enhances its recognition by autophagy receptor p62, ultimately leading to TBK1 autophagic degradation. Furthermore, an avirulent Δ*gra4* strain (ME49Δ*ompdc*/*gra4*) is constructed for tumor immunotherapy due to its ability to enhance IFN‐I production. Earlier vaccination with ME49Δ*ompdc*/*gra4* confers complete host resistance to the tumor compared with the classical ME49Δ*ompdc* treatment. Notably, ME49Δ*ompdc*/*gra4* vaccination induces a specific CD64^+^MAR‐1^+^CD11b^+^ dendritic cell subset, thereby enhancing T cell anti‐tumor responses. Overall, these findings identify the negative role of *T. gondii* GRA4 in modulating host IFN‐I signaling and suggest that GRA4 can be a potential target for the development of *T. gondii* vaccines and tumor immunotherapy.

## Graphical abstract

*T. gondii* effector GRA4 suppresses host harmful IFN‐I via degrading TBK1 through selective autophagy, which renders this parasitic protein a candidate to develop therapies against toxoplasmosis. Moreover, a vaccine designed by knocking out GRA4 in attenuated *T. gondii* boosts host IFN‐I responses and further recruits the IFN‐I dependent dendritic cells to prevent tumor growth. This study emphasizes a parasitic brake of host IFN‐I signaling and its potential in treating IFN‐I related diseases.

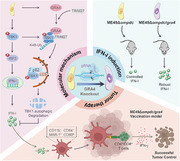

## 1 Introduction

*Toxoplasma gondii* (*T. gondii*) is an obligate intracellular parasite that infects a wide range of animals and causes zoonotic infections in an estimated one‐third of the human population globally.^[^ ^1^ ^]^ Upon *T. gondii* infection, toll‐like receptors (TLRs), nod‐like receptors, cyclic GMP‐AMP synthase (cGAS), absent in melanoma 2 (AIM2), and other host pattern recognition receptors (PRRs) detect pathogen‐associated molecular patterns (PAMPs). These include profilin, cyclophilin, dense granule (GRA), rhoptry (ROP), and GPI‐anchored (GPI‐A) proteins which are secreted by *T. gondii* to activate multiple immune signaling pathways,^[^ ^2^ ^]^ which can potentially transform this parasite from a villain of the peace to a potential anti‐*T. gondii* candidate or anti‐tumor warrior.

Host‐pathogen interactions exist extensively at various stages of *T. gondii* infection. GRA proteins secreted from the parasites could not only mediate the acquisition of host nutrients, but also form a translocon that delivers GRA effectors into the host cytosol, helping *T. gondii* to overcome the barrier of the parasitophorous vacuole (PV) membranes. In addition to their vital role in parasite development, increasingly studies indicate that several GRA proteins are also essential in controlling host immune responses.^[^ ^3^ ^]^ GRA4 is a protein found in the lumen of the PV and interacts with GRA6 to form the mature vacuole network during *T. gondii* invasion.^[^ ^4^ ^]^ This protein has been widely used, in combination with other antigens or adjuvants, for vaccine development. DNA vaccines containing GRA4 have been reported to achieve a 62% survival rate against acute *T. gondii* challenge.^[^ ^5^ ^]^ Previous studies have primarily focused on the phenotypes and vaccine treatment effects of GRA4. However, the underlying mechanism by which GRA4 regulates immune responses still needs to be further explored.

*T. gondii*‐induced host immune responses are mainly dependent on the cyst stage. In the acute stage of parasite invasion, the rapid replicating asexual cysts activate innate immune responses to limit the parasites' rapid parasite expansion in turn. However, to evade the immune system, *T. gondii* differentiates into slow‐growing, asexual cysts, which are the semi‐dormant form within muscle cells and neurons and have a lifelong existence. The latent *T. gondii* cysts can be reactivated and become dangerous in individuals whose immune system is impaired such as those with HIV/AIDS, those undergoing organ transplants or chemotherapy. As the first line of defense toward *T. gondii* infection, the innate immune system dictates the outcome of toxoplasmosis. Consequently, enhancing the innate immune response can effectively eradicate *T. gondii* at an early stage, thereby preventing the potential for covert infection during the chronic phase.

Previous studies suggested the negative role of type I interferon (IFN‐I) signaling in anti‐*T. gondii* immunity.^[^ ^6^ ^]^ The TANK‐binding kinase1 (TBK1) is the crucial kinase involved in the TLR‐, cGAS‐STING‐ and RIG‐I‐like receptors (RLR)‐MAVS‐mediated IFN‐I signaling pathway. This pathway plays an important role in antiviral and antitumor immunity,^[^ ^7^ ^]^ however the role of TBK1 and its regulatory mechanisms has not yet been explored in toxoplasmosis. Emerging studies have revealed that selective autophagy contributes to the degradation of TBK1 induced by viral proteins to inhibit IFN‐I signaling activation.^[^ ^8^ ^]^ Whether parasitic effectors could also act similarly on TBK1 remains unclear. Therefore, investigating the relationship between *T. gondii* effectors and TBK1 would be beneficial in explaining why IFN‐I production is often limited compared to IFN‐γ during toxoplasmosis, and for further modification of *T. gondii* for IFN‐I‐based anti‐tumor therapy.^[^ ^9^ ^]^

In this study, we found that mice infected with ME49Δ*gra4* exhibited a higher IFN‐I production and caused worse survival, along with severe pathology, when compared to the mice infected with WT ME49. However, the greater virulence of ME49Δ*gra4* failed to be embodied in the interferon α/β receptor knockout (IFNAR KO) mice. These results elucidate a previously unknown protective role of GRA4 to the host during toxoplasmosis. Furthermore, we present ME49Δ*ompdc*/*gra4*, a safe and effective tumor vaccine developed from *T. gondii* tachyzoites. Due to the lack of GRA4, a negative regulator that hijacks host TBK1, vaccination with ME49Δ*ompdc*/*gra4* results in stronger IFN‐I responses and induces IFN‐I‐dependent CD11b^+^CD64^+^MAR‐1^+^ dendritic cells (DCs), which in turn activate potent T cell responses to prevent tumor growth. The opposite roles of GRA4 in regulating anti‐*T. gondii* and anti‐tumor immunity are mediated by the same signaling pathway. This mechanism involves GRA4 interacting with activated TBK1, which promotes TBK1 autophagic degradation via K48‐linked polyubiquitination at Lys251/372 by the E3 ligase TRIM27, serving as a recognition signal for the cargo receptor p62. Overall, these findings improve our comprehension of the interplay between host‐pathogen interactions, selective autophagy, and innate immunity, yielding valuable insights for the development of biotherapies targeting cancers and other diseases.

## 2 Results

### 2.1 Toxoplasma Effector GRA4 Restrains Host IFN‐I Signaling

**Figure 1:**
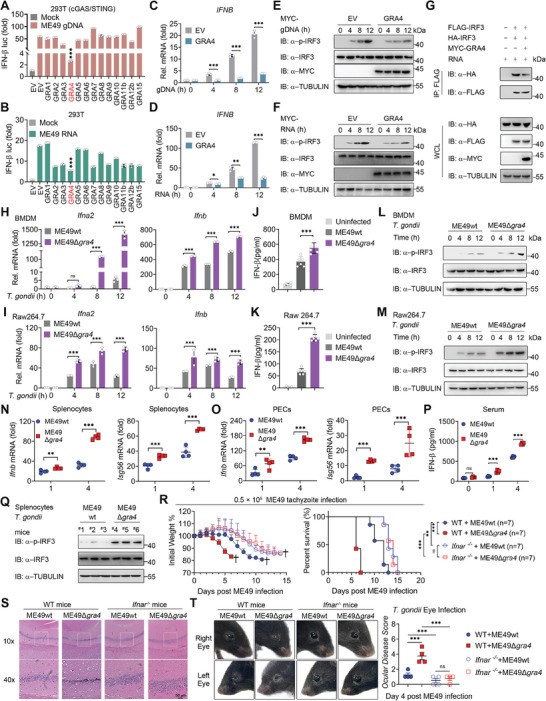
Dense granule 4 (GRA4) negatively regulates host type‐I interferon (IFN‐I) signaling and promote anti‐*T. gondii* immunity in an IFN‐I dependent manner. A) Luciferase activity in 293T cells (cGAS+STING stably expressing) transfected with a luciferase reporter for IFN‐β luc, together with empty vector (EV) or different types of dense granule (GRA) proteins, followed by treatment with or without ME49 genomic DNA (gDNA). Results are expressed relative to renilla luciferase activity. B) Luciferase activity in 293T cells transfected with a luciferase reporter for IFN‐β luc, together with EV or different types of GRA proteins, followed by treatment with or without ME49 RNA. Results are expressed relative to renilla luciferase activity. C,D) qPCR analysis of *IFNB* in 293T cells transfected with MYC‐GRA4 or EV followed by ME49 gDNA and ME49 RNA treatment at indicated time points. E,F) Immunoblotting analysis of total and phosphorylated IRF3 in 293T cells transfected with MYC‐GRA4 or EV followed by ME49 gDNA and ME49 RNA treatment at indicated time points. G) Immunoblotting analysis of 293T cells transfected with various combinations of plasmid encoding FLAG‐IRF3, HA‐IRF3, and MYC‐GRA4, and treated with ME49 RNA, followed by immunoprecipitation (IP) with anti‐Flag beads. H,I) qPCR analysis of *Ifna2* and *Ifnb* in bone marrow‐derived macrophages (BMDMs) or Raw264.7 cells followed by ME49wt or ME49∆*gra4* (MOI = 5) infection at indicated time points, is shown. J,K) ELISA of IFN‐β cytokines production in the supernatants of BMDMs or Raw264.7 cells, which were infected with ME49wt or ME49∆*gra4* (MOI = 5) for 24 h. L,M) Immunoblotting analysis of total and phosphorylated IRF3 in BMDM or Raw264.7 followed by ME49wt or ME49∆*gra4* (MOI = 5) infection at indicated time points. N,O) qPCR analysis of *Ifnb* and *Isg56* in Splenocytes and PECs from wild type (WT) C57 mice infected with ME49wt or ME49∆*gra4* (1 × 10^6^) on day 1 and day 4. P) ELISA of IFN‐β cytokine production in serum from wide type C57 mice at day 0, 1 and 4, which were infected with ME49wt or ME49∆*gra4* (1 × 10^6^). Q) Immunoblotting analysis of total and phosphorylated IRF3 in splenocytes from WT C57 mice infected with ME49wt or ME49∆*gra4* (1 × 10^6^). ^#^1 to ^#^6 represent different mice, respectively. R) Body weights and survival rates of wide type (*n* = 7) and *Ifnar^−/‐^* C57 mice (n = 7) infected with ME49wt or ME49∆*gra4* (0.5 × 10^5^) respectively as indicated, are shown. S) Representative photomicrographs of hippocampal HE staining in the four groups. HE staining shows morphological changes in the WT and *Ifnar^−/−^* mice hippocampus, which were infected with ME49wt or ME49∆*gra4* (0.5 × 10^5^). T) Representative micrographs of right and left eyes from WT and *Ifnar^−/−^* mice followed by ME49wt or ME49∆*gra4* (0.5 × 10^5^) infection on day 4, and the statistical analysis of ocular disease score, are as shown. Data with error bars are represented as means ± SD. Each panel is a representative experiment of at least three independent biological replicates. luc: luciferase, IP: immunoprecipitation, WCL, whole cell lysis, IB: immunoblotting. ^*^ *p* < 0.05, ^**^ *p* < 0.01, ^***^ *p* < 0.001, and ns (not significant) as determined by unpaired Student's t test, two‐way ANOVA, or the log rank test.

We first investigated the potential *T. gondii* GRA proteins that regulate IFN‐I signaling, via a luciferase reporter assay for IFN‐β in 293T cells, after transfection with GRA proteins and *T. gondii* nucleic acid stimulation. We found that GRA4 suppressed RLR‐ and cGAS‐STING‐mediated IFN‐I signaling to a significant extent (**Figure** **1**A,B). Moreover, GRA4 overexpression robustly suppressed transcription of interferon β (*IFNB*), interferon stimulating gene 15 (*ISG* *15*) and *ISG56* induced by parasitic genomic DNA (gDNA) or RNA (Figure 1C,D; Figure S1A,B, Supporting Information). Next, we observed that transfection of GRA4 restrained the activation of IFN‐I signaling induced by various stimuli, including *T. gondii* nucleic acids, poly(dA:dT), and poly(I:C), in a dose‐dependent manner (Figure S1C–F, Supporting Information). Brain cells are known to be the target of *T. gondii*, and we found overexpression with GRA4 can also suppress *T. gondii* nucleic acids‐induced transcription of *Ifnb*, *Isg15*, and *Isg56* in the BV2 cells, a mouse derived microglial cell line (Figure S1G,H, Supporting Information). Similarly, ectopic expression of GRA4 in 293T cells treated with *T. gondii* nucleic acids greatly inhibited the phosphorylation of IRF3, which is a hallmark of IFN‐I signaling activation (Figure 1E,F). Phosphorylated IRF3 dimerizes and translocates into the nucleus, where it turns on the expression of type I IFNs.^[^ ^10^ ^]^ To test whether GRA4 impacts the dimerization of IRF3, we transfected GRA4 into 293T cells and performed a co‐immunoprecipitation (co‐IP) assay. The results indicated that IRF3 formed dimerization post *T. gondii* RNA stimulation, and this combination considerably decreased in the presence of GRA4 (Figure 1G).

To further examine the role of endogenous GRA4 of *T. gondii* during parasite infection, we generated a GRA4‐knockout (KO) *T. gondii* based on wild strain ME49 (ME49wt), using the CRISPR/Cas9 system with single guide RNAs (sgRNAs), which we named ME49Δ*gra4*, and RT‐PCR was employed to verify the knockout effect (Figure S2A–C, Supporting Information). We next performed a plaque test, which showed growth rate of parasites, and found the plaque size of ME49Δ*gra4* showed no statistical significance compared to ME49wt, suggesting that loss of GRA4 did not affect *T. gondii* tachyzoites' proliferation (Figure S2D, Supporting Information). We next utilized the transgenic parasites to infect mouse primary bone marrow‐derived macrophages (BMDMs) and Raw264.7 mouse macrophage cell lines. As expected, GRA4 deletion led to a noteworthy increase in *Ifna2*, *Ifnb*, and *Isg56* transcription but had no effect on the proinflammatory factor *Il‐6*. (Figure 1H,I; Figure S2E,F, Supporting Information). Similarly, the protein levels of IFN‐β and the phosphorylation of IRF3 were also enhanced in mouse macrophages infected with ME49Δ*gra4*, compared to ME49wt (Figure 1J–M), which indicates that GRA4 inhibits *T. gondii* ME49 induced IFN‐I expression in the parasite‐infected macrophages in vitro. Together, these results demonstrate that the GRA4 protein acts as a suppressor of host IFN‐I signaling.

### 2.2 GRA4 Promotes Host Anti‐*T. Gondii* Immunity in an IFN‐I‐Dependent Manner

Because of the major effect of GRA4 on IFN‐I signaling in vitro, we analyzed a model system in which we challenged wide type (WT) mice with ME49wt or ME49Δ*gra4* to evaluate the ability of the GRA4 in modulating the anti‐parasite immunity in vivo, and mice were sacrificed at day 1 and day 4 post infection for immune analysis. We consistently observed enhanced IFN‐I‐related gene and protein expression in splenocytes, peritoneal cells (PECs), and serum of ME49Δ*gra4* infected WT mice (Figure 1N–Q; Figure S2G, Supporting Information). Our previous work has suggested that IFN‐I negatively regulates anti‐*T. gondii* ME49 immunity.^[^ ^6^ ^]^ Therefore, we aimed to evaluate whether ME49Δ*gra4* could cause severe toxoplasmosis by promoting IFN‐I production. Our initial findings revealed that ME49Δ*gra4* infection resulted in a higher parasite load in the splenocytes and PECs of WT mice (Figure S1H,I, Supporting Information). In addition, ME49Δ*gra4* infected WT mice experienced greater weight loss and reduced survival rates than those infected with ME49wt (Figure 1R; Figure S2J, Supporting Information). Moreover, WT mice infected with ME49Δ*gra4* exhibited more severe damage to the hippocampus and ocular diseases compared to ME49wt‐infected mice (Figure 1S,T). Nevertheless, the observed differences in toxoplasmosis outcomes and infection phenotypes resulting from GRA4 depletion in ME49 were not present when infecting *Ifnar* ^−/−^ mice (Figure 1R–T). Collectively, these results suggest GRA4 deficient *T. gondii* leads to a more severe IFN‐I‐dependent pathology in vivo, demonstrating that GRA4 functions as a protective effector for the host in battling toxoplasmosis.

### 2.3 GRA4 Specifically Interacts with Active TBK1

**Figure 2:**
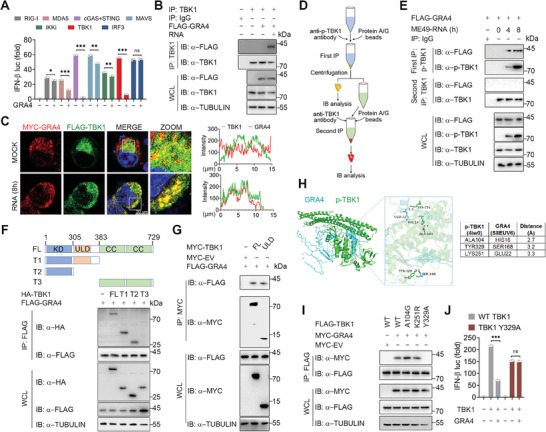
GRA4 specifically interacts with activated TANK Binding Kinase 1 (TBK1). A) Luciferase activity in 293T cells transfected with a luciferase reporter for IFN‐β luc, and FLAG tagged ‐RIG‐I, ‐MDA5, ‐MAVS, ‐cGAS plus STING, ‐TBK1, ‐IKKi, ‐IRF3(5D), together with or without GRA4. Results are expressed relative to renilla luciferase activity. B) Immunoblotting analysis of 293T cells transfected with FLAG‐GRA4 or ‐EV, and treated with ME49 RNA for 8 h, followed by IP with anti‐TBK1. C) Representative confocal images of 293T cells overexpressing MYC‐GRA4 and FLAG‐TBK1. Cells are treated with parasitic RNA for 8 h. Nuclei are stained with DAPI. Scale bars, 20 µm. The intensity analysis is next to the image. D) Schematic of two‐step IP in 293T cells. Protein extracted from 293T cells are immunoprecipitated through anti‐p‐TBK1 and protein A/G as first IP, using anti‐TBK1 and protein A/G antibodies as second IP. E) Immunoblotting analysis of 293T cells transfected with FLAG‐GRA4, and treated with ME49 RNA for indicated time, followed by IP with anti‐p‐TBK1 and second IP with anti‐TBK1. F,G) Immunoblotting analysis of 293T cells transfected with FLAG‐GRA4 and truncations of HA‐ or MYC‐TBK1, followed by IP with anti‐FLAG or anti‐MYC beads. H) Protein structure and molecular docking maps of p‐TBK1 and GRA4. I) Immunoblotting analysis of 293T cells transfected with various combinations of plasmid encoding FLAG‐WT TBK1, ‐A104G mutant, ‐K251R mutant or ‐Y329A mutant of TBK1 and MYC‐GRA4, followed by IP with anti‐FLAG beads. J) Luciferase activity in 293T cells, transfected with a luciferase reporter for IFN‐β luc, WT TBK1 and Y329A mutant of FLAG‐TBK1, together with or without MYC‐GRA4. Results are expressed relative to renilla luciferase activity. luc: luciferase, IP: immunoprecipitation, WCL, whole cell lysis, IB: immunoblotting. Data with error bars are represented as means ± SD. Each panel is a representative experiment of at least three independent biological replicates. ^*^ *p* < 0.05, ^**^ *p* < 0.01, ^***^ *p* < 0.001, and ns (not significant) as determined by unpaired Student's t test.

The members of the RLR family primarily detect various types of cytoplasmic pathogenic RNA, while the cytoplasmic pathogenic DNA is mainly sensed by cGAS, both kinds of nucleic acids sensing are responsible for the induction of type I interferon.^[^ ^11^ ^]^ To investigate the molecular mechanism underlying the suppression of IFN‐I signaling caused by GRA4, we performed a screen using dual luciferase reporter assay. The results indicated that overexpression of GRA4 significantly reduced the activity of IFN‐β and ISRE promoters, triggered by co‐transfecting plasmids expressing RIG‐I, MDA5, MAVS, cGAS plus STING, TBK1, or IKKi, except for IRF3, suggesting GRA4 inhibits the IFN‐I signaling upstream of IRF3, most likely by targeting at TBK1 (**Figure** **2**A; Figure S3A, Supporting Information). Of note, the co‐immunoprecipitation (co‐IP) assay revealed an interaction between ectopically expressed GRA4 and cell‐intrinsic TBK1 following parasitic RNA stimulation (Figure 2B; Figure S3B, Supporting Information). Moreover, the confocal immunofluorescence assay demonstrated that GRA4 rarely co‐localized with TBK1 in resting state cells, but parasitic RNA stimulation largely enhanced the co‐localization between GRA4 and TBK1 (Figure 2C).

To explore why GRA4 did not bind to TBK1 in the resting cells, we performed a complementary binding test that employed a two‐step co‐IP to separate phosphorylated TBK1 (p‐TBK1) from non‐phosphorylated TBK1 (Figure 2D). Interestingly, the co‐IP assay showed an increase in the association between p‐TBK1 and GRA4 after parasitic RNA stimulation, while there was no interaction between non‐phosphorylated TBK1 and GRA4 (Figure 2E). Similarly, the exogenous co‐IP assay also indicated that GRA4 interacted strongly with WT TBK1, but only weakly with TBK1 S172A, a mutant that abolishes the autophosphorylation of TBK1 (Figure S3C, Supporting Information).

The domain mapping analysis conducted using TBK1 truncation mutants revealed that GRA4 could bind with both the kinase domain (KD) and ubiquitin‐like domain (ULD) of TBK1, but not with the coiled‐coiled (CC) domain of this protein (Figure 2F,G). Furthermore, we performed molecular docking studies to predict multiple sites on p‐TBK1, including A104, Y329, and K251, which may be responsible for its binding ability with GRA4 (Figure 2H). Subsequently, we generated corresponding mutants to investigate the specific site of TBK1 that interacts with GRA4. Consistent with the Co‐IP assay results that the TBK1 Y329A mutant failed to bind to GRA4 (Figure 2I), the luciferase reporter assay also showed GRA4 could not inhibit the activation of IFN‐β that the TBK1 Y329A mutant triggered (Figure 2J), which identifies Y329 in the ULD domain of TBK1 as the crucial site for GRA4 binding to TBK1. Collectively, these results suggest that GRA4 interacts specifically with phosphorylated TBK1 at Y329.

### 2.4 GRA4 Promotes the Autophagic Degradation of Activated TBK1 in a p62‐Dependent Manner

**Figure 3:**
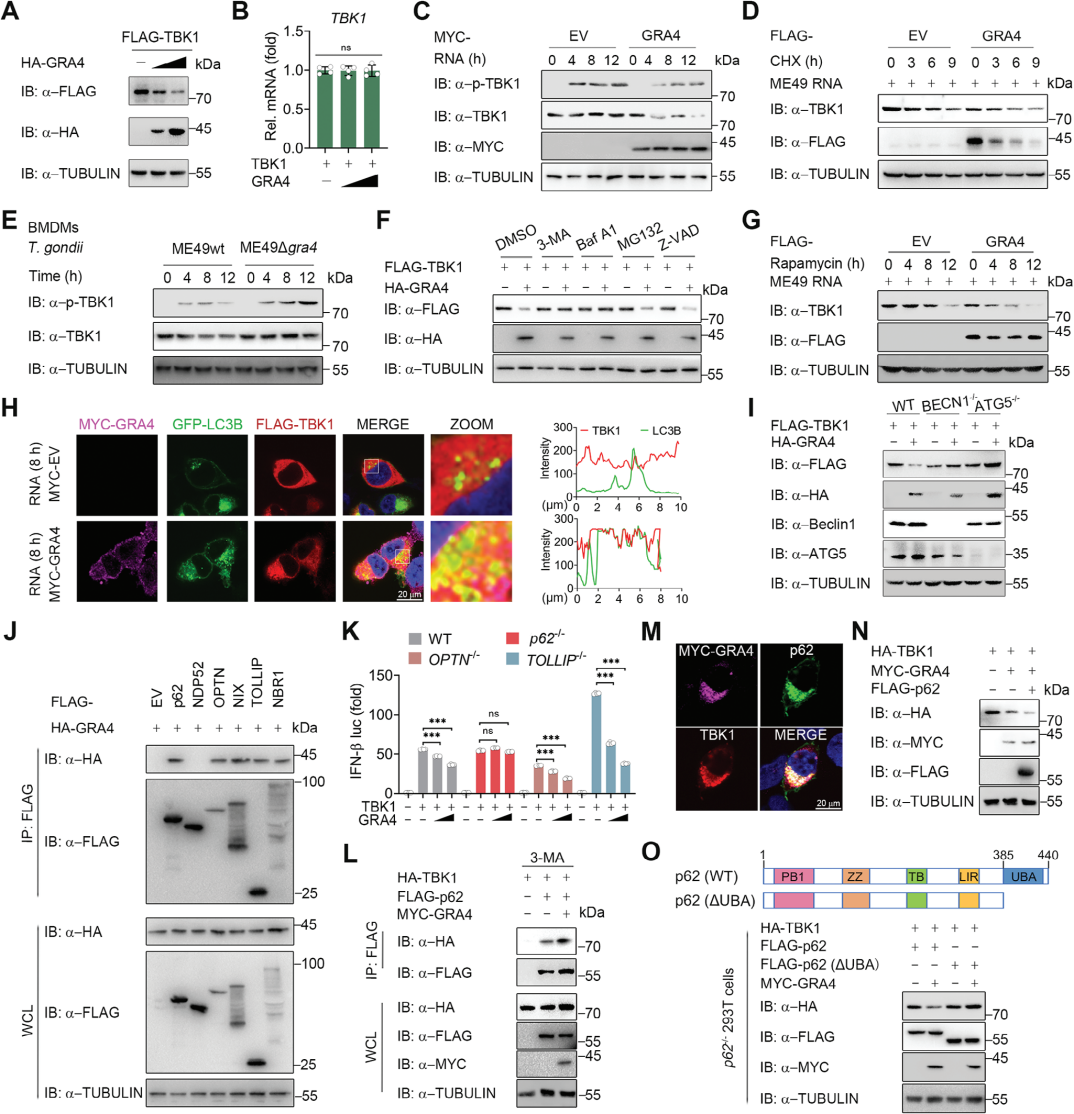
GRA4 induces the autophagic degradation of TBK1 in a Sequestosome 1 (SQSTM1)/p62 dependent manner. A,B) Immunoblotting and qPCR analysis of TBK1 protein and mRNA level extracts of 293T cells transfected with FLAG‐TBK1, HA‐EV or increasing amounts of HA‐GRA4. C) Immunoblotting analysis of total and phosphorylated TBK1 in 293T cells transfected with MYC‐EV or ‐GRA4, followed by treatment with ME49 RNA at indicated time points. D) Immunoblotting analysis of total TBK1 in 293T cells transfected with FLAG‐EV or ‐GRA4, pre‐stimulated by ME49 RNA for 8 h, and then treated with cycloheximide (CHX) (100 µg/mL) for indicated time points. E) Immunoblotting analysis of total and phosphorylated TBK1 in BMDMs followed by ME49wt or ME49*∆gra4* (MOI = 5) infection at indicated time points. F) Immunoblotting analysis of 293 T cells transfected with FLAG‐TBK1, together with HA‐EV or ‐GRA4, followed by treatments of 3‐MA (10 mm), bafilomycin A1 (Baf A1) (0.2 µm), MG132 (10 µm), and Z‐VAD (50 µm) for 6 h respectively. G) Immunoblotting analysis of total TBK1 in 293T cells transfected with FLAG‐EV or ‐GRA4, pre‐stimulated by ME49 RNA for 8 h and then treated with rapamycin (250 nM) for indicated time points. H) Representative confocal images of 293T cells overexpressing FLAG‐TBK1, GFP‐LC3B, together with MYC‐EV or GRA4. Cells are treated with parasitic RNA for 8 h. Nuclei are stained with DAPI. The intensity analysis is next to it. Scale bars, 20 µm. I) Immunoblotting analysis of WT, *BECN1* KO, and *ATG5* KO 293T cells transfected with FLAG‐TBK1, together with HA‐EV or HA‐GRA4. J) Immunoblotting analysis of 293T cells transfected with FLAG‐EV, ‐p62, ‐NDP52, ‐OPTN, ‐NIX, ‐TOLLIP, or ‐NBR1, and HA‐GRA4, followed by IP with anti‐FLAG beads. K) Luciferase activity in WT, *p62* KO, *OPTN* KO or *TOLLIP* KO 293T cells transfected with a luciferase reporter for IFN‐β‐luc, FLAG‐TBK1, HA‐EV or increasing amounts of HA‐GRA4, is shown. L) Immunoblotting analysis of 293T cells transfected with HA‐TBK1, FLAG‐p62 and MYC‐ GRA4, followed by treatment with 3‐MA (10 mm), and then IP with anti‐FLAG beads. M) Representative confocal images of 293T cells overexpressing FLAG‐TBK1, HA‐p62, together with MYC‐GRA4. Scale bars, 20 µm. N) Immunoblotting analysis of 293T cells transfected with various combinations of plasmid encoding HA‐TBK1, MYC‐GRA4, and Flag‐p62, is shown. O) Immunoblotting analysis of *p62* KO 293T cells transfected with HA‐TBK1, FLAG‐p62 or ‐p62ΔUBA, and MYC‐GRA4 or ‐EV. luc: luciferase, IP: immunoprecipitation, WCL, whole cell lysis, IB: immunoblotting. Data with error bars are represented as means ± SD. Each panel is a representative experiment of at least three independent biological replicates. ^***^ *p* < 0.001 and ns (not significant) as determined by unpaired Student's t test.

To further investigate the mechanisms by which GRA4 regulates TBK1 through their interaction, we transfected TBK1 along with an increased amount of GRA4 in 293T cells and BV2 cells. The results showed that GRA4 significantly decreased the protein level of TBK1 in a dose‐dependent manner, but did not affect the mRNA level (**Figure** **3**A,B; Figure S4A, Supporting Information). As we had determined that GRA4 specifically targets activated TBK1, we next attempted to explore whether GRA4 particularly degrades p‐TBK1 when IFN‐I signaling is activated by parasitic nucleic acids. As anticipated, GRA4 only reduced TBK1 protein level in the presence of *T. gondii*‐DNA or ‐RNA stimulation (Figure 3C; Figure S4B, Supporting Information). Consistently, we found that overexpressed GRA4 accelerates the degradation of endogenous TBK1 in 293T cells treated with cycloheximide (CHX), a protein translation inhibitor (Figure 3D), which suggested that GRA4 drives the PTM of TBK1. Consistent with the Figure S3C (Supporting Information), GRA4 also failed to degrade inactivated TBK1 S172A mutant (Figure S4C, Supporting Information). Signal peptides (SP) are short peptides located at the N‐terminal of proteins, which act as identifiers indicating the protein secretion pathway and its destination.^[^ ^12^ ^]^ Domain mapping analysis showed that GRA4 contains a 20 amino acid SP (Figure S4D, Supporting Information). To determine whether the SP of GRA4 affects its interaction with TBK1, we constructed a GRA4 mutant without the SP (GRA4ΔSP) and found that GRA4ΔSP could still degrade TBK1 and inhibit TBK1‐induced IFN‐I activation (Figure S4E,F, Supporting Information).

To further check whether GRA4 could degrade TBK1 during *T. gondii* cellular infection, we compared the intrinsic protein level change of both TBK1 and p‐TBK1 in macrophages infected with ME49wt and ME49Δ*gra4*, and found ME49wt infection caused the degradation of TBK1, while knockout of GRA4 in ME49 rescued the protein reduction of TBK1 and enhanced TBK1 phosphorylation induced by *T. gondii* infection (Figure 3E; Figure S4G, Supporting Information).

To identify the degradation system contributing to GRA4‐mediated TBK1 degradation, we separately applied the autophagy inhibitor 3‐methyladenine (3‐MA), bafilomycin A1 (Baf A1), proteasome inhibitor MG132, or pan‐Caspase inhibitor Z‐VAD to treat 293T cells overexpressing TBK1 and GRA4. Immunoblotting analysis showed that only autophagy inhibitors, 3‐MA and Baf A1, could block GRA4‐induced TBK1 degradation, while MG132 and Z‐VAD had no effect (Figure 3F). Besides, overexpressed GRA4 aggravated the autophagic degradation of activated TBK1 (Figure 3G). LC3 is the optimal marker to experimentally track the autophagosome since it is the only essential macroautophagy protein present in the autophagosome post‐completion.^[^ ^13^ ^]^ To examine the association between TBK1 and LC3, we utilized immunoblotting and immunofluorescence assays, and observed that GRA4 enhanced the interaction between TBK1 and LC3 in ME49 RNA‐stimulated cells (Figure 3H; Figure S4H, Supporting Information). To verify the crucial role of autophagy in degrading activated TBK1 by GRA4, we employed transgenic cells lacking the autophagy initiators, ATG5 or BECLIN1, in the 293T cells. Our results indicated that GRA4‐induced TBK1 degradation and IFN‐I inhibition were abrogated in these transgenic cells (Figure 3I; Figure S4I, Supporting Information). Hence, our findings suggest that GRA4 induces the autophagic degradation of TBK1.

The cargo receptors play a key role in identifying protein complexes, aggregates, or entire organelles with specific signals to transport them to autophagosomes for degradation.^[^ ^14^ ^]^ Next, we sought to identify the cargo receptor responsible for the autophagic degradation of TBK1 induced by GRA4. We conducted a co‐IP analysis to investigate the interaction between GRA4 and cargo receptors, including NDP52, p62, OPTN, Nix, TOLLIP, and NBR1. Our results demonstrated that GRA4 could interact with all cargo receptors except NDP52 (Figure 3J). Previous research indicated that TBK1 directly binds to p62, NDP52, OPTN, and TOLLIP.^[^ ^6^ ^]^ Therefore, we focused on p62, OPTN, and TOLLIP, which were found to interact with both GRA4 and TBK1. We discovered that TBK1 stability and IFN‐I signaling activation are no longer impaired by GRA4 in p62 KO 293T cells, while deletion of OPTN or TOLLIP did not alter the outcome (Figure 3K; Figure S4J,K, Supporting Information). Moreover, we found that p62, GRA4, and TBK1 formed a complex and GRA4 strengthened the interaction between TBK1 and p62 (Figure 3L,M). Additionally, we found that p62 was involved not only in the autophagic degradation of TBK1 (Figure S4L, Supporting Information), but also accelerated GRA4‐mediated degradation of TBK1 (Figure 3N). p62 is a widely studied cargo receptor to co‐aggregate with and degrade ubiquitinated substrates.^[^ ^15^ ^]^ The C‐terminal ubiquitin‐associated (UBA) domain of p62 is essential for recognizing the ubiquitinated proteins for degradation.^[^ ^16^ ^]^ To ascertain the role of p62 in GRA4‐induced TBK1 autophagic degradation, we overexpressed GRA4 with either WT p62 or p62ΔUBA mutant in p62‐deficient 293T cells, and found that GRA4 failed to induce TBK1 degradation when restored with the p62ΔUBA mutant in p62 KO cells (Figure 3O). Collectively, these results suggest that p62 is required for GRA4‐induced TBK1 autophagic degradation.

### 2.5 GRA4 Recruits TRIM27 for Catalyzing the K48‐Linked Ubiquitination of TBK1 at K251 and K372

**Figure 4:**
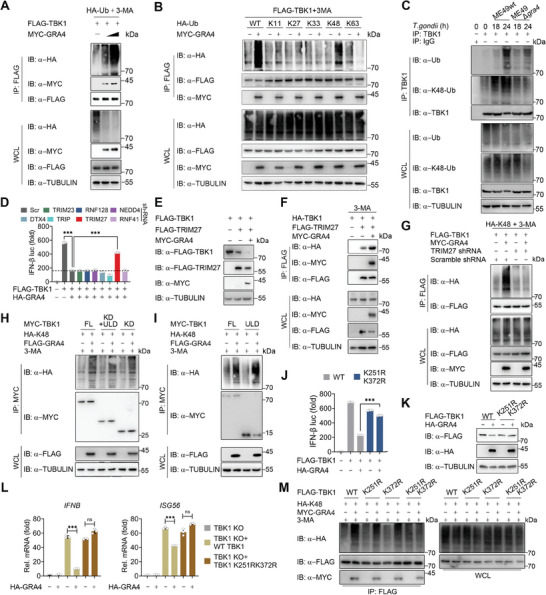
GRA4 recruits Tripartite Motif protein 27 (TRIM27) to catalyze TBK1 K48‐ubiquitination at Lys251 and Lys372 sites. A) Immunoblotting analysis of 293T cells transfected with various combinations of plasmid encoding FLAG‐TBK1, HA‐Ub, and MYC‐GRA4, followed by treatment with 3‐MA (10 mm), and then IP with anti‐FLAG beads. B) Immunoblotting analysis of 293T cells transfected with FLAG‐TBK1, HA‐WT‐, ‐K11‐, ‐K27‐, ‐K33‐, ‐K48‐ or ‐K63‐linked Ub, together with MYC‐EV or ‐GRA4, followed by treatment with 3‐MA (10 mm), and then IP with anti‐FLAG beads. C) Immunoblotting analysis of extracts of BMDMs infected with ME49wt or ME49*∆gra4* (MOI = 5) for the indicated time points, followed by IP with anti‐TBK1. D) Luciferase activity in 293T cells transfected with a luciferase reporter for IFN‐β‐luc, Scr shRNA or E3 ligases specific shRNAs for 24 h, followed by transfected with FLAG‐TBK1, together with HA‐EV or ‐GRA4 for 24 h. E) Immunoblotting analysis of 293T cells transfected with various combinations of plasmid encoding HA‐TBK1, FLAG‐TRIM27, and MYC‐GRA4, is shown. F) Immunoblotting analysis of 293T cells transfected with HA‐ TBK1, FLAG‐TRIM27, and MYC‐GRA4, followed by treatment with 3‐MA (10 mM), and then IP with anti‐FLAG beads. G) Immunoblotting analysis of 293T cells transfected with *Scramble* shRNA or TRIM27 specific shRNA for 24 h, then transfected with various combinations of plasmid encoding FLAG‐TBK1, HA‐K48‐Ub, and MYC‐GRA4 or EV, followed by treatment of 3‐MA for 6 h, and then IP with anti‐FLAG beads. H,I) Immunoblotting analysis of 293T cells transfected with MYC‐Full Length or truncations of TBK1, HA‐K48‐Ub, together with or without FLAG‐GRA4, followed by treatment with 3‐MA (10 mm), and then IP with anti‐MYC beads. J) Luciferase activity in 293T cells transfected with a luciferase reporter for IFN‐β‐luc, FLAG‐WT or K251R/K372R mutant of TBK1, together with HA‐EV or ‐GRA4, is shown. K) Immunoblotting analysis of 293T cells transfected with FLAG‐WT or K251R/K372R mutant of TBK1, together with HA‐EV or ‐GRA4, is shown. L) qPCR analysis of *IFNB* and *ISG56* in *TBK1* KO 293T cells transfected with FLAG‐WT or ‐K251R/K372R mutant of TBK1, together with HA‐EV or GRA4, is shown. M) Immunoblotting analysis of 293T cells transfected with FLAG‐WT or ‐K251R/K372R mutant of TBK1, HA‐K48‐Ub, together with or without HA‐GRA4, followed by treatment with 3‐MA (10 mm), and then IP with anti‐FLAG beads. luc: luciferase, IP: immunoprecipitation, WCL, whole cell lysis, IB: immunoblotting. Data with error bars are represented as means ± SD. Each panel is a representative experiment of at least three independent biological replicates. ^***^ *p* < 0.001 and ns (not significant) as determined by unpaired Student's t test.

Ubiquitination is a widespread PTM that regulates various stages of autophagy.^[^ ^17^ ^]^ To investigate the potential relationship between GRA4 and TBK1 ubiquitination, we overexpressed GRA4 in 293T cells treated with 3‐MA to inhibit TBK1 degradation. The results indicated that GRA4 upregulates TBK1 ubiquitination in a dose‐dependent manner (**Figure** **4**A). A variety of ubiquitin chains are attached as selective labels on protein aggregates and dysfunctional organelles, thus promoting their autophagy‐dependent degradation.^[^ ^17^ ^]^ K27‐, K33‐, K48‐, and K63‐linked polyubiquitination have been reported to regulate the function or stability of TBK1.^[^ ^18^ ^]^ To determine which ubiquitin chain is responsible for GRA4‐mediated degradation of TBK1, we analyzed the changes in different ubiquitin chain conjugations to TBK1 in the presence or absence of GRA4. Co‐IP and immunoblotting analysis showed that GRA4 specifically enhanced the K48‐linked polyubiquitination of TBK1 (Figure 4B). Furthermore, we observed that ME49 infection results in increased total ubiquitination and K48‐linked polyubiquitination of TBK1 in BMDMs over a prolonged period, but this tendency was abandoned during ME49Δ*gra4* infection (Figure 4C). Taken together, these findings suggest that GRA4 promotes the K48‐linked polyubiquitination of TBK1.

E3 ubiquitin ligases mediate the transfer of ubiquitin from an E2 ubiquitin‐conjugating enzyme to specific substrate proteins at the end of a three‐enzyme cascade, which is essential for promoting substrate proteins ubiquitination and degradation.^[^ ^19^ ^]^ Previous studies have identified several E3 ligases, including DTX4, TRIP, TRIM27, TRIM23, RNF41, RNF128, and NEDD4, that participate in the ubiquitination of TBK1. Subsequently, we silenced the expression of the E3 ligases known to relate to TBK1 with shRNAs and observed that the knockdown of TRIM27 largely rescued the impaired TBK1‐activated IFN‐I signaling induced by GRA4, but not the other E3 ligases (Figure 4D). As TRIM27 was previously reported to promote TBK1 degradation via the ubiquitin‐proteasome system (UPS), we aimed to investigate if it could also contribute to its autophagic degradation. Indeed, TRIM27 facilitated the autophagic degradation of TBK1 induced by starvation (Figure S5A, Supporting Information). Consistently, both lysosome and autophagy inhibitors rescued the TRIM27‐induced degradation of TBK1 (Figure S5B, Supporting Information). Additionally, GRA4 was found to accelerate TRIM27‐mediated TBK1 degradation, and enhance the interaction between TRIM27 and TBK1 (Figure 4E,F). To further examine the role of TRIM27 in the K48‐linked polyubiquitination of TBK1, we silenced TRIM27 and found that GRA4 no longer enhanced the K48‐linked polyubiquitination of TBK1 (Figure 4G). These findings highlight the vital role of TRIM27 in catalyzing K48‐linked polyubiquitination of TBK1 in the presence of GRA4.

The attachment of lysine residues within substrate proteins to the C‐terminus of Ub through an isopeptide bond is vital for the process of canonical ubiquitination.^[^ ^20^ ^]^ We first assessed the stability of each truncation of TBK1 and discovered that GRA4 induced degradation of both the KD and ULD domains, but not the CC domain of TBK1 (Figure S5C,D, Supporting Information). The ubiquitination of KD and ULD domains was also enhanced by GRA4 (Figure 4H,I). A previous study reported that TRIM27 induces TBK1 ubiquitination at K251 and K372 sites,^[^ ^21^ ^]^ which is consistent with our finding as the two sites are within the KD and ULD domain. We next verified whether these two sites are also essential for GRA4 to degrade TBK1 and inhibit IFN‐I signaling. As expected, the activation of IFN‐I signaling and TBK1 stability were not impaired upon mutation of these two sites, but not the other lysine residues within KD and ULD domains (Figure 4J,K; Figure S5E,F, Supporting Information). Consistently, the GRA4‐induced suppression of transcriptional level of *IFNB* and *ISG56* led by parasitic RNA was abolished in TBK1 K251R/K372R mutant‐reconstituted cells (Figure 4L). Moreover, GRA4 did not increase K48‐linked polyubiquitination chains on TBK1 K251R/K372R mutant (Figure 4M). Collectively, these results suggest that K251 and K372 sites in TBK1 are critical for TRIM27, which is recruited by GRA4, to catalyze the K48‐linked polyubiquitination of TBK1 for its autophagic degradation.

### 2.6 The Regulatory Role of GRA4 in Anti‐*T. Gondii* Immunity Relies on TBK1

As GRA4 plays such an elaborate regulatory role targeting TBK1, we next sought to link the in vitro molecular mechanism to in vivo anti‐*T. gondii* immunity. According to previous publications, mice that lack TBK1 result in embryonic lethality.^[^ ^22^ ^]^ So, we here employed in vivo *Tbk1* small interfering RNA (siRNA) to verify that whether the regulatory role of GRA4 depends on TBK1 (Figure S6A,B, Supporting Information). Consistent with the above conclusion that GRA4 could promote anti‐*T. gondii* immunity in an IFN‐I dependent manner, we here also found ME49Δ*gra4* no longer exhibit stronger virulence, which was reflected by the weight loss, survival, and ocular toxoplasmosis, than ME49wt in the *Tbk1*‐slienced host (Figure S6C–E, Supporting Information). Generally, these results suggest TBK1‐IFN‐I axis is necessary for GRA4 to regulate anti‐*T. gondii* immunity.

### 2.7 Mice Vaccinated with ME49Δ*ompdc* Shows Improved Resistance to Tumors in an IFN‐I‐Dependent Manner

Increasing evidence indicates that non‐replicating avirulent *T. gondii* inhibits tumor growth and has been explored for tumor immunotherapy.^[^ ^23^ ^]^ However, there is still a need for improvement in safety and efficacy. Given IFN‐I responses from the host play a crucial role in all stages of tumorigenesis and progression,^[^ ^24^ ^]^ and abolishing GRA4 in ME49 enhances host IFN‐I responses by targeting TBK1, we hypothesized whether the value of GRA4 deficient *T. gondii* in enhancing IFN‐I production could be applied to host tumor immunotherapy. To ensure safety when using a “bug as a drug”, we created an avirulent, nonreplicating transgenic ME49 by abolishing OMPDC and LDH (hereafter ME49Δ*ompdc*). This strain is intended for immunotherapy and we established a comparative model system to evaluate the efficacy of two different *T. gondii*‐treatment approach in combating tumor growth. The first approach, referred to as “Vaccination”, involves pre‐infecting mice with ME49Δ*ompdc* on day 7 and day 1 prior to tumor implantation. In the second approach, named “Treatment”, mice were infected with ME49Δ*ompdc* on day 1 and day 7 following tumor implantation (Figure S7A, Supporting Information). As presented, earlier treatment of ME49Δ*ompdc* markedly suppressed tumor growth and prolonged survival time in mice bearing B16‐F10 or MC38 tumors compared to those receiving later treatment that could also suppress tumorigenesis moderately (Figure S7B–E, Supporting Information). Subsequently, we evaluated the immune indices of mice bearing tumors that received “Vaccination”. We first applied qPCR analysis to detect the transcriptional level of positive or negative regulators of anti‐tumor immunity. Vaccination with ME49Δ*ompdc* resulted in an elevated expression of positive genes, including *Ifng*, *Tnfa*, and *Il12*, while suppressing negative genes like *Il6*, *Pd1*, and *Pdl1*, in tumor or splenocytes compared to cells from tumor‐bearing mice treated with the PBS (Figure S7F,G, Supporting Information). Consistently, gene expression and FACS analysis revealed that ME49Δ*ompdc* vaccination significantly increased the percentage of CD4^+^ and CD8^+^ T cells in tumors and spleens, but not in tumor‐draining lymph nodes (TDLNs), while lowering the expression of PD‐1 in these splenic and intratumoral T cells (Figure S7H–L, Supporting Information). In addition, in vitro activation and intracellular cytokine staining showed an enhancement of IFN‐γ expressing CD4^+^ and CD8^+^ T cells in mice vaccinated with ME49Δ*ompdc* (Figure S7M,N, Supporting Information).

IFN‐I responses from the host play a crucial role in all stages of tumorigenesis and progression.^[^ ^24^ ^]^ However, it remains unclear whether IFN‐I is indispensable for *T. gondii‐*mediated anti‐tumor immunotherapy, particularly for the ME49Δ*ompdc* vaccination. We compared the tumor growth of WT, *Mavs* ^−/−^, *Tmem173* ^−/−^, and *Ifnar* ^−/−^ mice that were vaccinated with ME49Δ*ompdc*, and found the potent anti‐tumor efficiency of ME49Δ*ompdc* vaccination was dismissed in those IFN‐I signaling deficient mice (Figure S8A,B, Supporting Information), and ME49Δ*ompdc* vaccination no longer altered the expression of these tumor‐associated genes when mice lacked IFN‐I and downstream signaling (Figure S8C,D, Supporting Information). Consistently, the percentage of CD4^+^ and CD8^+^ T cells within tumors and spleens from the IFN‐I signaling deficient mice was comparable between the vaccinated and control group in *Ifnar* ^−/−^ mice (Figure S8E,F, Supporting Information). Collectively, these results suggest that ME49Δ*ompdc* vaccinated mice show improved resistance to tumors that rely on IFN‐I.

### 2.8 Mice Vaccinated with ME49Δ*ompdc/gra4* Exhibit Enhanced IFN‐I Responses and Further Inhibit Tumor Growth in the TBK1 Dependent Manner

**Figure 5:**
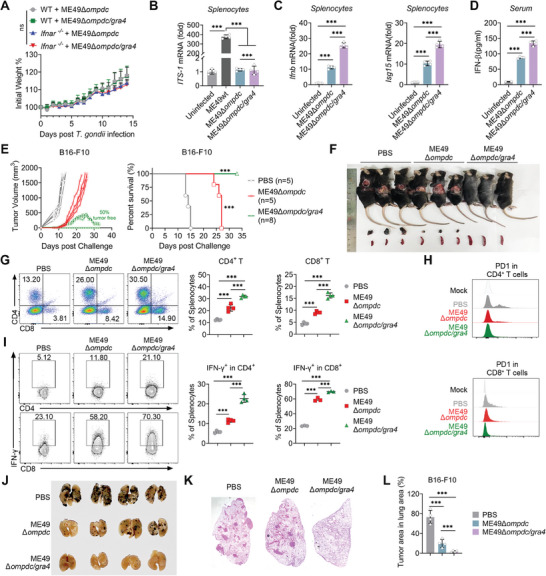
Mice vaccinated with ME49Δ*ompdc*/*gra4* activates stronger IFN‐I responses and completely inhibit tumor growth. A) Body weight change of WT and *Ifnar* ^−/‐^ mice vaccinated with ME49Δ*ompdc* or ME49Δ*ompdc*/*gra4*. B) qPCR analysis of *ITS‐1* gene expression in splenocytes from mice with or without ME49wt, ME49Δ*ompdc*, and ME49Δ*ompdc*/*gra4* infection. C) qPCR analysis of *Ifnb* and *Isg15* gene expression in splenocytes from mice with or without ME49Δ*ompdc* and ME49Δ*ompdc*/*gra4* immunization. D) ELISA of IFN‐β production in serum from mice with or without ME49Δ*ompdc* and ME49Δ*ompdc*/*gra4* immunization. E) Tumor growth (left) and survival curve (right) of WT mice vaccinated with ME49Δ*ompdc*, ME49Δ*ompdc*/*gra4* or PBS, followed by implanted B16‐F10 tumor cells. F) The size and location of the tumors detected in mice (top), and changes in the tumor or spleen (bottom) volume dissected from mice vaccinated with ME49*Δompdc*/*gra4*, ME49Δ*ompdc*, or PBS, followed by implanted B16‐F10 tumor cells. G) Representative flow plots (left) and histogram (right) of CD4^+^ and CD8^+^ T cells within splenocytes from mice vaccinated with ME49Δ*ompdc*, ME49Δ*ompdc*/*gra4*, or PBS, followed by implanted B16‐F10 tumor cells. H) Representative quantification of PD‐1 expression in CD4^+^ (top) and CD8^+^ (bottom) T cells from mice vaccinated with ME49Δ*ompdc*, ME49*Δompdc*/*gra4*, or PBS, followed by implanted B16‐F10 tumor cells. I) Representative plots (left) and histogram (right) of IFN‐γ of CD4^+^ T and CD8^+^ T cells within splenocytes from mice vaccinated with ME49Δ*ompdc*, ME49Δ*ompdc*/*gra4*, or PBS, followed by implanted B16‐F10 tumor cells. J) Macroscopic evaluation of B16‐F10 tumors metastasis in the lungs of mice treated with PBS (isotype control), ME49Δ*ompdc*, or ME49Δ*ompdc*/*gra4* via vaccination schedules, together with injecting intravenously (*i.v*.) with B16‐F10 cells. K) Representative histopathology images of lung in J). L) Percentage of lung area occupied by tumor lesions in mice shown in J). Data with error bars are represented as means ± SD. Each panel is a representative experiment of at least three independent biological replicates. ^***^ *p* < 0.001 and ns (not significant) as determined by unpaired Student's t test, two‐way ANOVA, or the log rank test.

Given the vital role of IFN‐I in ME49Δ*ompdc*‐induced anti‐tumor immunotherapy, and abolishing GRA4 in ME49 enhances host IFN‐I responses by targeting TBK1, we hypothesized that vaccination with ME49Δ*ompdc/gra4* could be more effective in preventing tumor growth. The virulence of the ME49Δ*ompdc/gra4* vaccine was first evaluated relative to ME49Δ*ompdc*. Our findings indicated that ME49Δ*ompdc/gra4* immunization did not affect the body weight of mice when compared to ME49Δ*ompdc* due to nonreplicability (**Figure** **5**A). Additionally, parasitic DNA was almost undetectable in the spleens of mice treated with either ME49Δ*ompdc* or ME49Δ*ompdc/gra4* at day 7 post‐infection (Figure 5B). In line with deleting GRA4 in ME49wt causing stronger IFN‐I responses within macrophages in vitro, ME49Δ*ompdc/gra4* treatment also induced increased IFN‐I signaling activation in the gene and protein level in vitro (Figure S9A–G, Supporting Information). We next examined the ability of ME49Δ*ompdc/gra4* vaccination to trigger IFN‐I production in vivo by qPCR and ELISA analysis and obtained the consistent conclusion (Figure 5C,D). Generally, these results imply that ME49Δ*ompdc/gra4* is a safe vaccine that augments host IFN‐I responses.

Next, we evaluated the effect of ME49Δ*ompdc/gra4* in anti‐tumor vaccination and found that immunization with ME49Δ*ompdc/gra4* mutants had greater effectiveness in resisting tumor growth and improving survival rates in tumor‐bearing mice when compared to ME49Δ*ompdc* vaccination (Figure 5E,F). These findings are in line with this vaccine's capability to induce stronger IFN‐I responses. Strikingly, vaccination using ME49Δ*ompdc*/*gra4* resulted in around 50% of the mice being tumor‐free, while the other 50% of tumor‐bearing mice eventually resolved the tumors (Figure 5E,F). We then compared the immunophenotyping of splenocytes isolated from ME49Δ*ompdc/gra4* vaccinated tumor‐free mice to those tumor‐bearing mice treated with either ME49Δ*ompdc* or PBS. FACS analysis revealed that ME49Δ*ompdc/gra4* vaccination led to a greater increase in the percentage of CD4^+^ and CD8^+^ T cells, a downregulation of PD‐1 expression on the surface of these cells, and an increase in IFN‐γ cytokines production in CD4^+^ and CD8^+^ T cells (Figure 5G–I). Additionally, compared to ME49Δ*ompdc*, vaccination with ME49Δ*ompdc*/*gra4* further reduced the burdens of lung tumors in mice injected with B16‐F10 cells intravenously (*i.v*.), indicating that this safe vaccine also holds promise in inhibiting pulmonary metastasis (Figure 5J–L). Collectively, these results provide compelling evidence of the potent protective effects of ME49Δ*ompdc/gra4* vaccination in preventing tumor growth.

### 2.9 The Anti‐Tumor Effect of ME49Δ*ompdc*/*gra4* Vaccination Relies on TBK1

As TBK1 is indispensable for GRA4 to regulate IFN‐I responses, we next sought to verify whether the anti‐tumor effect of ME49Δ*ompdc*/*gra4* relies on TBK1. Similarly, we employed *Tbk1* siRNA to knockdown TBK1 in vivo (Figure S10A, Supporting Information), and found that ME49Δ*ompdc*/*gra4* vaccination could not inhibit tumor growth and prolong host survival time when the host *Tbk1* was silenced (Figure S10B–D, Supporting Information). Consistently, ME49Δ*ompdc*/*gra4* vaccination no longer promoted the proportion of splenic CD4^+^ and CD8^+^ T cells (Figure S10E,F, Supporting Information), and the capability of ME49Δ*ompdc*/*gra4* vaccination in both promoting CD4^+^ and CD8^+^ T cells producing IFN‐γ (Figure S10G,H, Supporting Information) and decreasing PD‐1 expression on the T cell surface (Figure S10I, Supporting Information) were also neutralized in the *Tbk1*‐knockdown host. Generally, these results suggest that the anti‐tumor function of ME49Δ*ompdc/gra4* vaccination relies on TBK1.

### 2.10 ME49Δ*ompdc/gra4* Vaccination Leads to Expansion of IFN‐I Dependent CD64^+^MAR‐1^+^CD11b^+^ DCs and Effective Anti‐Tumor Immunity

**Figure 6:**
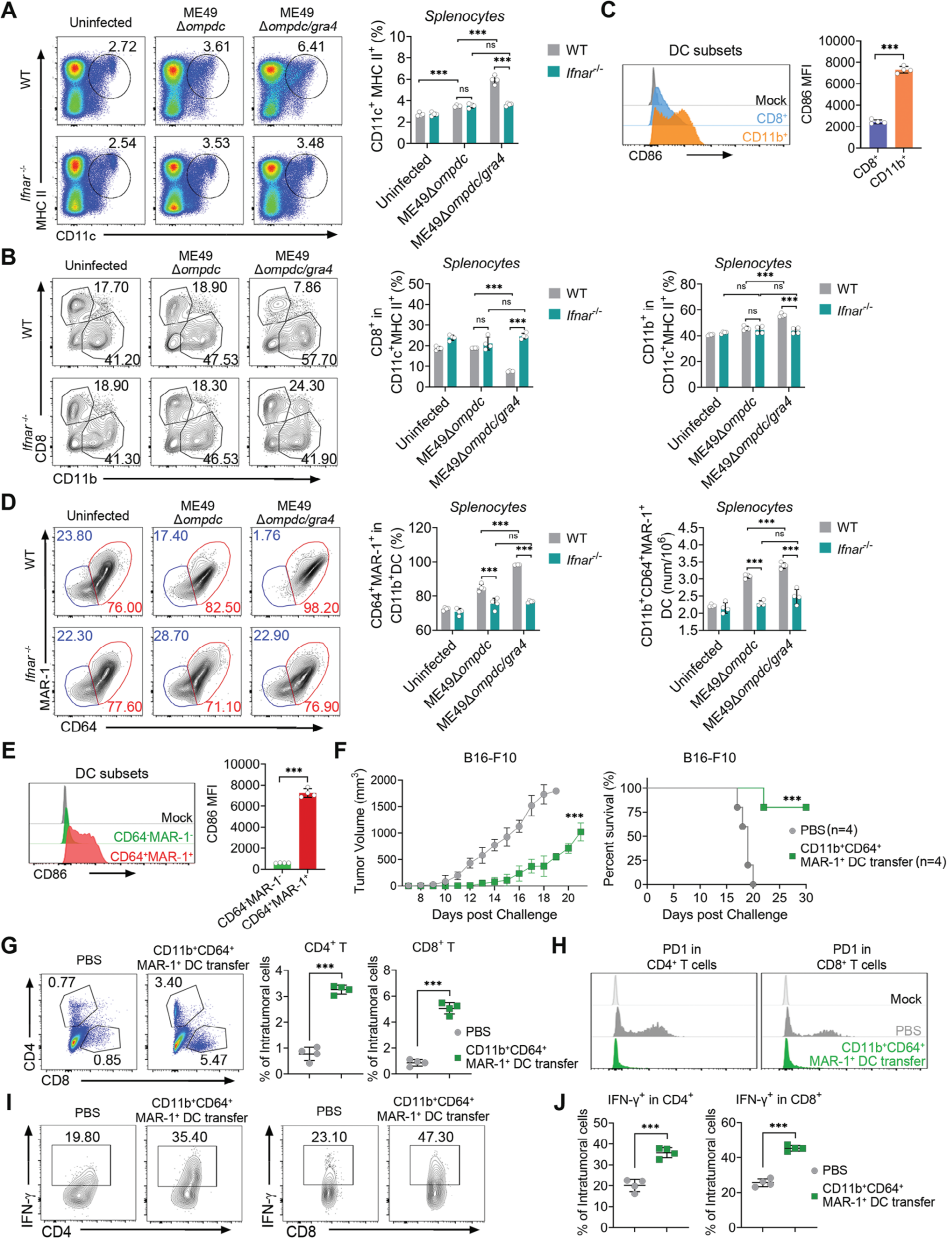
Mice immunized with ME49Δ*ompdc/gra4* activates IFN‐I dependent CD64^+^MAR‐1^+^CD11b^+^ dendritic cells (DCs) for efficient anti‐tumor immunity. A) Representative flow plots (left) and histogram (right) of CD11c^+^MHC‐II^+^ cells in splenocytes from WT or *Ifnar* ^−/−^ mice with or without ME49Δ*ompdc* or ME49Δ*ompdc*/*gra4* infection. B) Representative flow plots (left) and histogram (right) of CD8^+^ or CD11b^+^ cells in CD11c^+^MHC‐II^+^ cells of splenocytes from WT or *Ifnar* ^−/−^ mice with or without ME49Δ*ompdc* or ME49Δ*ompdc*/*gra4* infection. C) Representative flow cytometric analysis (top) and histogram (bottom) of CD86 median fluorescent intensity (MFI) expressed by CD8^+^ and CD11b^+^ DC subsets. D) Representative flow plots (left) and quantification (right) of CD64^+^MAR‐1^+^ in CD11b^+^ DCs of splenocytes from WT or *Ifnar* ^−/−^ mice with or without ME49Δ*ompdc* or ME49Δ*ompdc*/*gra4* infection. E) Representative flow cytometric analysis (top) and histogram (bottom) and of CD86 MFI expressed by CD64^−^MAR‐1^−^CD11b^+^ DC and CD64^+^MAR‐1^+^CD11b^+^ DC subsets. F) Tumor growth (left) and survival curve (right) of recipient mice with or without CD64^+^MAR‐1^+^CD11b^+^ DCs adoptive transfer, followed by implanted B16‐F10 tumor cells. G) Representative flow plots (left) and histogram (right) of CD4^+^ and CD8^+^ T cells within intratumoral cells from recipient mice with or without CD64^+^MAR‐1^+^CD11b^+^ DCs adoptive transfer, followed by implanted B16‐F10 tumor cells. H) Representative quantification of PD‐1 in CD4^+^ (left) and CD8^+^ (right) T cells in intratumoral cells from recipient mice with or without CD64^+^MAR‐1^+^CD11b^+^ DCs adoptive transfer, followed by implanted B16‐F10 tumor cells. I,J) Representative plots and histogram of IFN‐γ of CD4^+^ T (I) and CD8^+^ T (J) cells in intratumoral cells from recipient mice with or without CD64^+^MAR‐1^+^CD11b^+^ DCs adoptive transfer, followed by implanted B16‐F10 tumor cells. Data with error bars are represented as means ± SD. Each panel is a representative experiment of at least three independent biological replicates. ^***^ *p* < 0.001 and ns (not significant) as determined by unpaired Student's t test, two‐way ANOVA, or the log rank test.

ME49Δ*ompdc/gra4* vaccination could prevent tumor growth by activating T cells, and antigen‐presenting cells (APCs), including conventional DCs (cDCs, CD11c^+^MHC2^+^), plasmacytoid DCs (pDCs, CD11c^+^B220^+^), macrophages (CD11b^+^F4/80^+^), and monocytes (CD11b^+^Ly6C^hi^). And these cells are the major categories of innate cells that are involved in controlling spontaneous T cell responses against tumors.^[^ ^25^ ^]^ We next aimed to determine the APC responsible for T cell activation and tumor suppression induced by ME49Δ*ompdc/gra4*. We compared the proliferation of APCs sorted from splenocytes of ME49Δ*ompdc* or ME49Δ*ompdc/gra4* immunized WT or *Ifnar* ^−/−^ mice (Figure S11A Supporting Information). The results showed that ME49Δ*ompdc* immunization elevated the percentage of all four categories of APCs, while ME49Δ*ompdc/gra4* immunization additionally promoted the proliferation of cDCs, macrophages, and pDCs in the splenocytes from WT mice (**Figure** **6**A; Figure S11B–D, Supporting Information). Moreover, we observed that the proportion of cDCs was not further amplified by ME49Δ*ompdc/gra4* immunization in the splenocytes from *Ifnar* ^−/−^ mice, relative to the other APCs. This suggests that ME49Δ*ompdc/gra4* induced potent IFN‐I, primarily stimulating cDCs proliferation and enhancing anti‐tumor immunity.

The functions of various DC subsets in anti‐tumor immunity are different.^[^ ^26^ ^]^ According to the function and genetic basis, cDCs can be classified into CD8^+^ DC and CD11b^+^ DC subsets.^[^ ^27^ ^]^ As shown in Figure 6B, ME49Δ*ompdc/gra4* infection leads to increased differentiation of primary DCs into CD11b^+^ DCs in the spleens of WT mice, no such effect was observed in the *Ifnar* ^−/−^ mice. Additionally, we found ME49Δ*ompdc/gra4* induced CD11b^+^ DC subsets expressed significantly higher amounts of the DCs maturation marker CD86 compared to CD8^+^ DCs (Figure 6C). Therefore, we concluded that CD11b^+^ DCs are the specific target cells of IFN‐I induced by ME49Δ*ompdc/gra4* treatment.

A recent study reported that IFN‐I could induce the CD64^mid^MAR‐1^+^CD11b^+^ cDC2 subset, known as inf‐cDC2, which acquires characteristics typically defining cDC1 and macrophages during respiratory viral infection and allergy.^[^ ^28^ ^]^ Therefore we investigated whether IFN‐I could also induce a similar CD11b^+^ DC subset during ME49Δ*ompdc/gra4* immunization. By FACS analysis, we found that the *ompdc/gra4* double knockout *T. gondii* mutants caused the accumulation of CD64^+^MAR‐1^+^CD11b^+^ DCs, and there was no change in this subset between ME49Δ*ompdc* and ME49Δ*ompdc/gra4* infected *Ifnar* ^−/‐^ mice (Figure 6D; Figure S11E, Supporting Information). However, unlike respiratory viral infection and allergy, the expression level of CD64 in ME49Δ*ompdc/gra4* induced CD11b^+^MAR‐1^+^ DCs could not be clearly distinguished between middle and high levels ((Figure 6E; Figure S11E, Supporting Information). Besides, the ME49Δ*ompdc/gra4* induced IFN‐I dependent CD64^+^MAR‐1^+^CD11b^+^ DCs expressed notably higher amounts of CD86 compared to CD64^−^MAR‐1^−^CD11b^+^ DCs (Figure 6E). Collectively, these findings indicate that ME49Δ*ompdc/gra4* vaccination leads to a robust proliferation and maturation of the CD64^+^MAR‐1^+^CD11b^+^ DC subset.

To further investigate the potential of *T. gondii* ME49Δ*ompdc/gra4* induced CD64^+^MAR‐1^+^CD11b^+^ DCs in anti‐tumor activity, we established an in vivo adoptive transfer model. CD64^+^MAR‐1^+^CD11b^+^ DCs were sorted from splenocytes of ME49Δ*ompdc/gra4* immunized mice and then intravenously injected into the WT mice, and tumor cells were then implanted on the right flank of the recipient subsequently (Figure S12A, Supporting Information). As we hypothesized, mice transferred with CD64^+^MAR‐1^+^CD11b^+^ DCs showed significant tumor resistance and a longer survival time (Figure 6F). By evaluating the immune responses, we found that CD64^+^MAR‐1^+^CD11b^+^ DCs adoptive transfer increased the percentage, lowered PD‐1 expression, and promoted the capacity of producing IFN‐γ in both splenic and intratumoral CD4^+^ and CD8^+^ T cells (Figure 6G–J; Figure S12B–E, Supporting Information). Consistently, CD64^+^MAR‐1^+^CD11b^+^ DCs adoptive transfer also altered the expression of tumor‐associated genes toward an anti‐tumor status (Figure S12F,G, Supporting Information). Collectively, these findings suggest that vaccination with ME49Δ*ompdc/gra4* induces the CD64^+^MAR‐1^+^CD11b^+^ DC subset, ultimately leading to successful anti‐tumor immunotherapy.

## 3 Discussion

*Toxoplasma* effectors target host gene transcription and protein post‐transcriptional modification to modulate multiple signaling pathways and immune responses.^[^ ^3^, ^29^ ^]^ A number of GRA proteins, including GRA6, GRA7, GRA15, GRA16, GRA24, and GRA28, have been confirmed to participate in host‐microbe interaction. *T. gondii‐*induced IFN‐I negatively regulates host anti‐*T. gondii* immunity, and its production is often at a lower level compared to IFN‐γ. This suggests the possible presence of parasitic effectors that suppress IFN‐I signaling. Here, we report that GRA4 hijacks phosphorylated TBK1 and inhibits IFN‐I production through selective autophagy. Additionally, we observed that deleting GRA4 in ME49 leads to a more robust IFN‐I response, inspiring us to develop an attenuated GRA4‐deficient transgenic ME49 for use in anti‐tumor immunotherapy.

Unlike the parasitic negative regulators mentioned above which target host signaling to aid immune evasion, we have found that GRA4 is capable of suppressing IFN‐I responses detrimental to the host. At times, *T. gondii* infection may fail to trigger significant IFN‐I production when compared to IFN‐γ, indicating the presence of negative regulatory mechanisms for IFN‐I, in addition to the reported GRA15 and SOCS1.^[^ ^6^, ^9^, ^30^ ^]^ Our screening of the suppressing role of GRA protein families discovered that GRA4 can inhibit both cGAS‐ and RLR‐induced IFN‐I activation. TBK1 is the core kinase in the signaling pathways of cGAS/RLR and TLRs, which is stringently controlled by various PTMs in infectious diseases and cancer,^[^ ^31^ ^]^ but its role in toxoplasmosis is not well understood. Our study demonstrates that GRA4 triggers selective autophagic degradation of activated TBK1 through a complex mechanism. GRA4 binds to the Y329 residue at the ULD domain of phosphorylated TBK1, promoting TRIM27 to catalyze TBK1 K48‐polyubiquitination at K251 and K372 sites. The ubiquitinated TBK1 then acts as the cargo to be recognized by p62 and degraded via the autophagy‐lysosome pathway.

Autophagy is an important weapon that the host has developed to control *T. gondii*, which relies on two forms of immune responses: i) clearance of T. *gondii* by CD40‐mediated autophagy, ii) IFN‐γ‐induced clearance of *T. gondii* through selective autophagy proteins, such as ATG3, ATG7, and the ATG5‐ATG12‐ATG16L1 complex, which are necessary to recruit guanylate binding proteins (GBPs) and IRGs.^[^ ^32^ ^]^ However, here our findings demonstrate that the *T. gondii* effector GRA4 can manipulate host IFN‐I signaling via selective autophagy. GRA4 triggers and participates in the entire TBK1 autophagic degradation process, but its structural simplicity and lack of a classical cargo receptor motif or E3 enzyme activity require additional host factors for full functionality. Besides, GRA4 interacts with both cargo receptor p62 and E3 ligase TRIM27, enhancing the connection between TBK1 and them. This suggests that the crosstalk between the parasitic proteins and the host autophagic process is a crucial aspect of host‐microbe interactions. However, our in vitro *T. gondii* infection model mostly relied on the macrophages, and the exploration of molecular mechanism was employed with Human embryonic kidney (HEK) 293T cells and BV2 cells with ectopic expression of GRA4. It is challenging to fully replicate the systemic infection of *T. gondii*, as this parasite can infect nearly all nucleated cells. It would be meaningful for us to further study the role of GRA4 within a wide range of host cells, using specific antibody against endogenous GRA4.

During *T. gondii* infection, the parasites may cross the vascular endothelium to access human retina by at least three routes: in leukocyte taxis; as a transmigrating tachyzoite; and after infecting endothelial cells. Once the host is intraocularly infected with *T. gondii*, it would cause the ocular toxoplasmosis (OT), a retinitis‐almost always accompanied by vitritis and choroiditis, which remains a challenging clinical ocular disease.^[^ ^33^ ^]^ Besides, *T. gondii* can migrate to and enter the central nervous system (CNS) and establish a persistent infection in neural and other brain cells to cause the toxoplasic encephalitis (TE),^[^ ^34^ ^]^ which is previously been regard as the second most common AIDS‐related opportunistic infection of the CNS with high incidence and mortality rates.^[^ ^35^ ^]^ TE induced inflammatory responses may also severely damage or alter neuronal function.^[^ ^36^ ^]^ There are also many neuron cells within hippocampus that could regulate virtually all aspects of cellular and circuit function,^[^ ^37^ ^]^ and previous studies identified that *T. gondii* can cross the blood‐brain barrier and infect different regions of the brain including the hippocampus.^[^ ^38^ ^]^ More interestingly, *T. gondii* exhibits more preference to infect hippocampus.^[^ ^39^ ^]^ Our in vivo studies showed GRA4 deficient ME49 induces higher IFN‐I which positively correlated to the symptoms, including ocular diseases and brain damage, of toxoplasmosis. We confirm the virulence of ME49Δ*gra4* relied on IFN‐I by using *Ifnar* ^−/−^ mice, which is consistent with our previous studies and further substantiates the harmful role of IFN‐I during *T. gondii* infection.^[^ ^6^ ^]^ GRA4 has been confirmed in inhibiting IFN‐I production in our acute *T. gondii* infection model. However, *T. gondii* infection is a quite complex process, and the immune responses and prognosis are determined by several factors: i) the mouse strains: BALB/c mice were reported to be more resistant to *T. gondii* infection than C57BL/6 mice, which has previously been linked to enhanced immune response specifically via MHC gene expression;^[^ ^40^ ^]^ ii) the *T. gondii* strains: previous studies identified ROP5, ROP16 and ROP18 as the major virulence determinants in mice between three major parasitic strains, which also determines the host immune responses.^[^ ^1a^ ^]^ However, little is known about the differences in expression of GRA4 between three *T. gondii* strains, and whether GRA4 can also act as the determinant to affect virulence of different parasitic strains still needs further research; iii) the infection patterns: infection with *T. gondii* tachyzoites was used to model the acute infection, which was usually to be applied for the study of anti‐*T. gondii* innate immunity, while oral infection with cysts is often to be employed for chronic *T. gondii* infection. We here chose the acute infection model and mainly focused on the role of GRA4 in regulating IFN‐I signaling and anti‐*T. gondii* innate immunity, which are limited somehow, and the role of GRA4 in chronic infection and adaptive immunity still need further studied. So, there still needs to be more research effort in exploring the function of GRA4 in different pre‐clinical experimental models using different *T. gondii* strains, infection patterns, and various hosts, which would further provide more evidence to establish recombinant GRA4 as a candidate of great potential for *T. gondii* vaccine development. Moreover, certain autoimmune diseases, including systemic lupus erythematosus (SLE), Sjogren's syndrome, and systemic sclerosis, require precise control of IFN‐I responses,^[^ ^41^ ^]^ making GRA4 a potential therapeutic option for treating these diseases.

Conversely, IFN‐I is protective in anti‐viral and anti‐tumor immunity, so here we constructed attenuated GRA4 deficient ME49 (ME49Δ*ompdc*/*gra4*) and employed it for anti‐tumor immunotherapy. Emerging studies claimed that bacteria, viruses, and/or fungi are key actors in cancer immunotherapy, and can be engineered to treat metastases by modulating the host immune system.^[^ ^42^ ^]^ The transgenic *T. gondii* and related PAMPs have been widely applied in anti‐tumor therapy since 2013, and the mechanisms behind their efficient anti‐tumor effect are diverse.^[^ ^23^, ^43^ ^]^ Some studies not only support the role of *T. gondii* in inhibiting tumor growth, but also show long‐term protection against re‐challenged tumors, which reminds us to design a pre‐vaccinated strategy using ME49Δ*ompdc* to prevent tumor growth. As we showed, earlier treatment with ME49Δ*ompdc* showed enhanced capacity in resisting tumors compared to the later treatment. Additionally, it was found that pre‐vaccinated ME49Δ*ompdc* is ineffective in *Ifnar* ^−/‐^ mice, indicating the indispensability of IFN‐I in the process. Furthermore, pre‐vaccination with GRA4 deficient ME49Δ*ompdc* could further improve the anti‐tumor effect of ME49Δ*ompdc* by triggering stronger IFN‐I and inducing CD64^+^MAR‐1^+^CD11b^+^ DCs that are exceptionally activated in generating potent T cell responses producing IFN‐γ, which slows down and even completely inhibits tumor growth. Compared to the previous studies, our findings not only progress the approach to treating tumors, but also clarify the underlying mechanism.

Among different types of APCs, DCs have a potent ability to recognize pathogenic and tumor‐specific antigens, and they exhibit specialized antigen‐presenting functions, making them indispensable in both anti‐tumor and anti‐pathogen immunity.^[^ ^44^ ^]^ Moreover, DCs are closely correlated with IFN‐I as DCs are the primary sources of IFN‐I, and IFN‐I feedback regulates the maturation, migration, and differentiation of DCs. There are also similarities and differences in the mechanism for IFN‐I in regulating different DC subsets during anti‐tumor immunity. IFN‐I can not only induce intratumoral accumulation of CD8a^+^ DCs, but also control antigen retention and enhance the survival of CD8a^+^ DCs after the uptake of tumor apoptotic cells, which leads to cross‐priming.^[^ ^45^ ^]^ Besides, Ellen et al. reported that tumor cell‐derived IFN‐I can activate MHC class I‐dressed CD11b^+^ cDCs to promote protective anti‐tumor CD8^+^ T cell immunity, and they named this DC subset as ISG^+^ DC.^[^ ^46^ ^]^ In addition to promoting anti‐tumor immunity, IFN‐I induces a new DC subset called ifn‐cDC2 (CD11c^+^CD11b^+^CD26^+^CD64^mid^MAR‐1^+^), which acquires features of cDC1s and macrophages to orchestrate immunity against respiratory virus infection.^[^ ^28^ ^]^ Similarly, we also found ME49Δ*ompdc/gra4* immunization can expand the population of CD64^+^MAR‐1^+^CD11b^+^ DCs in an IFN‐I‐dependent manner. A recent study showed that RORγt agonist (8‐074) promotes monocyte‐derived dendritic cells through CXCL10 in cancers to enhance anti‐PD‐1 therapy.^[^ ^47^ ^]^ In order to determine whether the *T. gondii*‐induced CD64^+^MAR‐1^+^CD11b^+^ DCs could suppress tumor growth like the reported agonist, we administrated an in vivo adoptive transfer assay and confirmed the anti‐tumor ability of CD64^+^MAR‐1^+^CD11b^+^ DCs.

**Figure 7:**
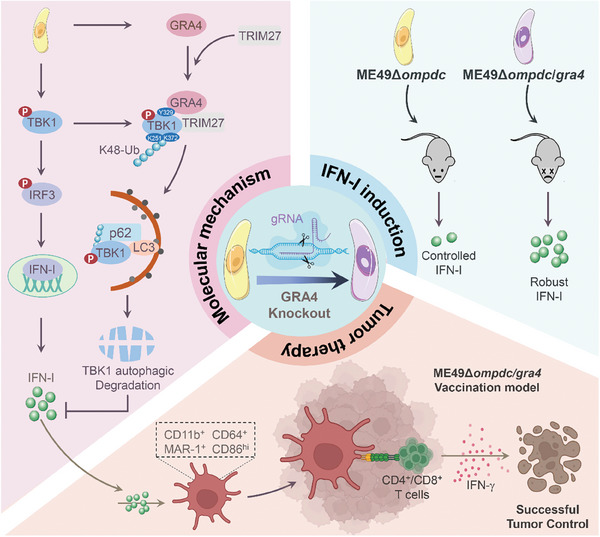
GRAPH ABSTRACT: The dual character of GRA4 in regulating anti‐*T. gondii* and anti‐tumor immunity via suppressing IFN‐I signaling. *Toxoplasma* effector protein GRA4 induces TRIM27‐p62‐dependent selective autophagic degradation of TBK1 to inhibit host IFN‐I responses. On one hand, GRA4 helps host to prevent severe toxoplasmosis. On the other hand, GRA4 limits anti‐tumor efficiency induced by attenuated *T. gondii*, and vaccination with the modified ME49Δ*ompdc/gra4* activates stronger IFN‐I production to promote the proliferation of CD64^+^MAR‐1^+^CD11b^+^ DCs and drive enhanced T cell responses, which confers host complete resistance to the tumor.

Collectively, GRA4 is an exogenous parasitic regulator that acts as an immune brake for host IFN‐I signaling by promoting selectively autophagic degradation of TBK1. While the absence of GRA4 in WT ME49 resulted in more severe pathology, the removal of GRA4 in attenuated ME49Δ*ompdc* enabled it to enhance its role in anti‐tumor immunity by boosting IFN‐I production and accumulating CD64^+^MAR‐1^+^CD11b^+^ DCs through our pre‐vaccination approach. We believe that both the GRA4 protein and attenuated ME49Δ*ompdc/gra4* can be applied as a potential treatment to facilitate clinical translation of microbial biochemicals (**Figure** **7**).

## 4 Experimental Section

### Mice

WT mice were purchased from Guangdong Medical Laboratory Animal Center. Transgenic mice including *Ifnar* ^−/−^, *Tmem173* ^−/−^, and *Mavs* ^−/−^ were laboratory kept. All mice experiments were approved by the Southern Medical University Animal Care and Use Committee (SMUL20201010). Mice (6‐8 weeks old) within experiments were age‐ and sex‐matched. Mice were housed under specific pathogen‐free (SPF) conditions with 50 to 60% humidity and daily cycles of 12 h of light at an ambient temperature of 21° to 23 °C.

### Transgenic ME49 Mutant Construction

GRA4‐specific CRISPR plasmid pSAG1‐Cas9‐sgGRA4 was generated by replacing the MIC3‐ targeting gRNA sequence in pSAG1‐Cas9‐sgMIC3 with a GRA4‐specific gRNA, following the protocols described previously.^[^ ^48^ ^]^ The GRA4 double knockout strains (patented) were constructed by co‐electroporating the pSAG1::Cas9‐U6::sg‐GRA4 plasmid and the 5H‐Loxp‐DHFR‐Loxp‐3H fragment into purified tachyzoites of the ME49 or ME49Δ*ompdc*Δ*ldh1* strains.^[^ ^49^ ^]^ Transfectants were selected with 1 µm pyrimethamine and single clones were identified by diagnostic PCRs. The plasmids used are listed in Appendix Table S1 (Supporting Information).

### T. gondii Infection Models

ME49wt and ME49Δ*gra4* strain were propagated intracellular in rat embryonic fibroblasts (REFs) cultured in complete Dulbecco's modified Eagle's medium (DMEM) (Corning, Cat#10‐013‐CVR) supplemented with 10% fetal bovine serum (FBS) (Vazyme, Nanjing, China), penicillin‐streptomycin (100 µg mL^−1^) (Gibco, Cat# 15140148). Tachyzoites were isolated and purified through a 5.0‐µm Nuclepore membrane (Sigma‐Aldrich, Cat#TMTP02500) and washed twice with PBS (Bio‐Channel, Nanjing, China).

For in vivo infection, WT or *Ifnar* ^−/−^ mice were intraperitoneally injected with 1×10^6^ ME49wt or ME49Δ*gra4* tachyzoites suspended in PBS for immunological index, like cytokine and related gene expression. For survival, weight change, simple neuro assessment of asymmetric impairment (SNAP), and parasite burden assay, WT or *Ifnar* ^−/−^ mice were intraperitoneally injected with 0.5×10^5^ ME49wt or MW49Δ*gra4* tachyzoites suspended in PBS. Mice were sacrificed and tissues were collected at indicated time points for immune analysis. The ocular disease score was determined as previously reported.^[^ ^50^ ^]^ Briefly, two observers performed ocular disease scoring (0‐5, 5 being severe) in a blinded fashion based on the following scoring system: 0‐no symptoms, 1‐mild symptoms with <20% eyelid shut, 2‐moderate symptoms with 20–50% shut, 3‐moderate symptoms with 50–80% shut, 4‐severe symptoms with >80% shut, 5‐eye completely shut with crusting. The damage of the hippocampus was measured by haematoxylin & eosin (H&E) staining of the brain (horizontal plane) that was performed by Servicebio of China.

For in vitro infection, mouse primary bone marrow‐derived macrophages (BMDMs) or peritoneal macrophages (PEMs) were challenged with ME49wt or MW49Δ*gra4* tachyzoites at MOI = 3 for indicated time points, then, cells and supernatant were collected for indicated analysis.

### Tumor Models

B16‐F10 or MC38 cells were washed and resuspended in PBS and injected subcutaneously on the flank of WT or indicated transgenic mice that were pre‐shaved (2×10^6^ cells per mouse in 200 µL PBS). Mice were monitored every day and tumors were measured by vernier caliper every day since the tumor appeared. Mice were sacrificed at indicated time points for immune analysis or when tumors reached 2000 cm^3^ of volume. To establish a melanoma pulmonary metastasis model, 5×10^5^ B16‐F10 cells resuspended in 200 µL PBS were intravenously (*i.v*.) injected, and mice with different treatments were sacrificed post ≈20 days to observe the lung tumor burdens.

### Attenuated T. Gondii Earlier or Later Treatment Model

Attenuated ME49Δ*ompdc* and ME49Δ*ompdc/gra4* mutants were propagated intracellularly in the REFs cultured in complete DMEM with an additional 250 µmol L^−1^ uracil. Tachyzoites were isolated and purified through a 5.0 µm Nuclepore membrane and washed twice with PBS. For earlier treatment, mice were pre‐challenged with ME49Δ*ompdc* intraperitoneally at Day 7 and Day 1 before the tumor was implanted. For later treatment, mice were infected with ME49Δ*ompdc* at Day 1 and Day 7 after the tumor had been implanted.

### In Vivo siRNA Knockdown of Tbk1

*Tbk1* siRNA (Santa Cruz, Cat#sc‐39059) or *Scr* siRNAs (Santa Cruz, Cat#sc‐37007) were intravenously injected in mice at indicated time points: i) in the *T. gondii* infection model, siRNAs were injected at Day 0, 4, 8 post *T. gondii* infection; ii) in the tumor model, siRNAs were injected at Day −7, −1 post ME49Δ*ompdc*/*gra4* vaccination. Besides, Entranster in vivo RNA transfection reagent (Engreen Biosystem, Cat#18668‐11‐1) was used as a vehicle for siRNA delivery according to the manufacturer's instructions.

### Cell Isolation and Culture

HEK293T, A549, THP‐1, BV2, B16‐F10 and REF were obtained from the American Type Culture Collection (ATCC). Cell lines mentioned above were maintained in complete DMEM supplemented with 10% FBS, penicillin‐streptomycin (100 µg mL^−1^). BMDMs and PEMs were isolated as previously reported.^[^ ^51^ ^]^ Briefly, mouse bone marrow cells were isolated from the tibia and femur and cultured in the conditional medium [90% DMEM, 10% FBS, 20% M‐CSF (20 ng mL^−1^) (R&D SYSTEMS, Cat# 416‐ML), penicillin‐streptomycin (100 µg mL^−1^)] in 24‐well plate (Promth, China) for 5 days to generate BMDMs. Mouse PEMs were acquired from ascites of indicated mice, which were injected intraperitoneally with 4% Brewer thioglycolate medium (BD Biosciences, Cat# 211716) for three consecutive days before sacrifice, then cold PBS was injected into the peritoneum of sacrificed mice, and fluid containing PEMs was aspirated from the peritoneum after the shake and collected after centrifugation at 800 rpm for 5 min.

### Cell Lines Generation via CRISPR/Cas9

Single‐guide RNA (sgRNA) sequences were designed via the website (http://www.e‐crisp.org/E‐CRISP/designcrispr.html), and the primer sequences were listed in the Appendix Table S2 (Supporting Information). sgRNA primers were annealed and cloned into lenti‐sgV2 vector to get *TBK1*‐, *Tollip*‐, or *OPTN*‐ targeting vectors and confirmed by sequencing. Next, these vectors were transfected into 293T cells for 48 h, and then the exchange of the supernatant with fresh culture medium contained puromycin (5 mg mL^−1^) (Sigma‐Aldrich, Cat# 540411). The live cells were screened out post‐48 h and subjected to WB analysis to verify the knockout efficiency.

### shRNA Construction

Indicated E3 ligase shRNA sequences were designed by the website (https://rnaidesigner.thermofisher.com/rnaiexpress/sort.do), and oligos of sequences with the highest rank were annealed and cloned into PLKO.1‐Puro vector and then confirmed by sequencing.

### Plasmid Construction and Transfection

Plasmids and their domain constructs were cloned into the pcDNA3.1 vector with FLAG‐/HA‐/MYC‐ tag for transient expression using In‐Fusion HD Cloning kits (Takara, Cat# 639650). The site‐directed mutagenesis (SDM) was administrated by MutanBEST Kit (Takara, Cat# R401) to generate relative mutants. HEK293T cells transfection were performed using StarFect Transfection Reagent (Genstar, Cat# C101) or Lipofectamine 2000 (Invitrogen, Cat# 11668019) according to the protocols recommended by the manufacturer.

### Cell Treatment

For inducing IFN‐I signaling activation, HEK293T cells were transfected with poly(I:C) (2 µg mL^−1^) (InvivoGen, Cat# tlrl‐picw), poly(dA:dT) (2 µg mL^−1^) (InvivoGen, Cat# tlrl‐patn), parasitic gDNA (2 µg mL^−1^), or parasitic RNA (2 µg mL^−1^) for indicated time points. HEK293T cells were treated with CHX (100 µg mL^−1^) (Selleck, Cat# S7418) to block protein synthesis. Rapamycin (250 nm) (MedChem Express, Cat# HY‐10219) was used for autophagy inducement. 3‐MA (10 mm) (Selleck, Cat# S2767) or Baf A1 (0.2 µM) (Selleck, Cat# S1413) was used to inhibit autolysosome‐ or lysosome‐mediated protein degradation respectively, and MG132 (10 µm) (Ambeed, Cat# A181909) inhibited proteasome‐mediated protein degradation, and Z‐VAD (50 µm) (Selleck, Cat# S7023) inhibited caspase‐mediated protein degradation.

### Isolation of ME49 gDNA and RNA

Isolation of *T. gondii* nucleic acids was performed as previously described.^[^ ^6^ ^]^ Briefly, tachyzoites were collected and purified with a 0.5 µm filter to avoid the influence of cell debris. Parasites were collected after filtration through centrifugation at 2000 g for 10 min, and the lysate was incubated with buffer A (150 mm NaCl, 25 mm EDTA, 10% SDS, and protein kinase) overnight. gDNAs were isolated using phenol‐chloroform extraction, and RNAs were isolated using TRIzol reagent (Invitrogen, Cat # 15596026CN).

### Luciferase and Reporter Assays

HEK293T cells (1×10^5^) were plated in 24‐well plates and transfected with plasmids encoding the IFN‐β or ISRE luciferase reporter (Firefly luciferase) and pRL‐TK (Renilla luciferase), together with different plasmids as indicated, or co‐treated with various stimulation. Samples were collected at 24 to 36 h after transfection, and luciferase activity was measured using the Dual‐Luciferase Reporter Assay Kit (Promega, Cat#1910) performed with a Luminoskan Ascent luminometer (Thermo Fisher Scientific). The activity of firefly luciferase was normalized by that of Renilla luciferase to obtain relative luciferase activity.

### Enzyme‐Linked Immunosorbent Assay (ELISA)

IFN‐β in cell supernatants and mice serum was measured with the Mouse IFN‐beta DuoSet ELISA kit (R&D SYSTEMS, Cat# DY8234‐05) following the assay procedure. Absorbance was detected at 450 nm by the Multiskan FC (Thermo Fisher Scientific).

### RNA Extraction and qRT‐PCR

Total RNA was extracted from splenic, tumor, lymph node, or stimulated cells through the TRIzol reagent (Invitrogen, Cat # 15596026CN) and then subjected to generate the complementary DNA (cDNA) via reverse transcription using a Starscript II first‐stand cDNA synthesis kit (GenStar, Cat# A211). Quantitative real‐time PCR was performed on QuantStudio 6 flex (Thermo Fisher Science) using a RealStar green power mixture (GenStar, Cat# A313) with primers as listed in Appendix Table S3 (Supporting Information).

### Molecular Docking

Molecular docking was performed using HDOCK (http://hdock.phys.hust.edu.cn/). The structure of p‐TBK1 (PDB code 4iw0) and GRA4 (predicated by AlphaFold using Uniprot ID S8EUV6 ^[^ ^52^ ^]^) were employed for model building, and the targeted amino acid residues were determined according to the interaction distance within 5Å. Structures and maps in the figures were rendered with PyMOL (The PyMOL Molecular Graphics System, v.4.0, Schrödinger).

### Immunoprecipitation and Immunoblot Assays

Cells were lysed by RIPA buffer (Merck, Cat# 20–188). For endogenous IP, whole cell lysates were treated with indicated antibodies overnight and then incubated with protein A/G beads (MedChem Express, Cat# HY‐K0202) for 4 to 6 h. For exogenous IP, whole‐cell lysates were only incubated with anti‐FLAG agarose beads (Sigma‐Aldrich, Cat# A2220) or anti‐MYC agarose beads (Alpalifebio, Cat# KTSM1306) overnight. Immunoprecipitates were eluted with 2×SDS loading buffer after washing five times with RIPA buffer, and boiled for 10 min, and next resolved by SDS‐PAGE. Proteins were transferred to a polyvinylidene difluoride membrane (Sigma‐Aldrich, Cat# 03010040001). The membranes were blocked with 5% (w/v) reagent‐grade nonfat milk and further incubated with the antibodies with universal antibody diluent (NCM Biotech), which are listed in Appendix Table S4 (Supporting Information), diluted with BSA (Sangon Biotech, Cat# A600903). EMD Millipore Luminata Western HRP Chemiluminescence Substrate was used for protein detection for all blots.

### Immunofluorescence and Confocal Microscopy

HEK293T cells were transfected with indicated plasmids for 48 h, and then washed three times using PBS, followed by fixation with 4% paraformaldehyde (Meilunbio, Cat# MA0192) (diluted in PBS) for 20 min and permeabilization with 0.2% Triton X‐100 (Sangon Biotech, Cat# A600198) for 20 min, block with 5% goat serum (Sangon Biotech, Cat# E510009) for 30 min at room temperature. The samples were then stained with the indicated primary antibodies as listed in Appendix Table S4 (Supporting Information) overnight at 4 °C, followed by incubation with fluorescent dye–conjugated secondary antibodies for 1 h at room temperature. The nuclei were counterstained with DAPI dihydrochloride (MedChem Express, Cat# HY‐D0814) for 5 min before being subjected to confocal microscopy observation. And the images were analyzed via ZEN (Carl Zeiss AG).

### Flow Cytometry and Cell Sorting

Antibodies applied in flow cytometry analysis are shown in Appendix Table S5 (Supporting Information). For cellular surface staining, mouse splenocytes or tumor cells were stained at 4 °C in RPMI (Corning, Cat# 10‐040‐CV) containing 2% FBS post using anti‐mouse CD16/CD32 to block FcR, and were incubated with specific antibodies for 30–60 min. For intracellular IFN‐γ staining, splenocytes were restimulated by Cell Stimulation Cocktail (plus protein transport inhibitors) (Invitrogen, Cat# 00‐4970‐93) within complete RPMI supplemented with 10% FBS, penicillin (100 U mL^−1^), and streptomycin (100 µg mL^−1^) for 8 h at 37 °C, then incubated with anti‐CD4 and anti‐CD8a for surface staining. Following fixation (Invitrogen, Cat# 00‐8222‐49) and permeabilization (TONBO, Cat# TNB‐1213), the splenocytes were stained with anti‐IFN‐γ monoclonal antibody. All samples were operated on BD LSRFortessa cytometer (BD sciences). The data were analyzed via FlowJo X software (Tree Star). For DCs sorting, the surface staining of total splenocytes from infected mice was performed as described above under sterile conditions, and targeted cells were acquired and sorted into RPMI containing 10% FBS, 1% penicillin/streptomycin, and 1 × HEPES using a BD FACSAria III.

### Adoptive Transfer

CD11b^+^CD64^+^MAR‐1^+^ DCs were isolated from splenocytes of WT mice that were vaccinated with ME49Δ*ompdc*/*gra4* at Day 4 post vaccination. Then, 2.5 × 10^6^ isolated cells were intravenously injected into each recipient mouse at Day 3 before tumors were implanted, and the tumor bearing mice with or without CD11b^+^CD64^+^MAR‐1^+^ DCs adoptive transfer were subsequently subjected to observe and analysis.

### Statistical Analysis

The graphical abstract was created with BioRender.com. All statistical analyses were performed using GraphPad Prism 9.4.1 (GraphPad software, inc.). All data are shown as mean ± SD. Statistical analyses were performed by unpaired Student's t test, one way ANOVA, or by the log rank test with ^*^ *p* < 0.05, ^**^ *p* < 0.01, ^***^ *p* < 0.001, and ns (not significant).

## Conflict of Interest

X.Y., Z‐R.L, and Z.Q.H. have filed a provisional patent (application number: 202410318945.1) related to the technology described in this work to China National Intellectual Property Administration. The other authors declare no conflict of interest.

## Author Contributions

Z.Q.H. and Y.F.Z. contributed equally to this work. Y.X., Z‐R.L., and Z.Q.H. performed the experiment design. Z.Q.H. and Y.F.Z. performed major experiments, performed data analysis, and wrote the original draft. Y.X., K.Z., Z.X.H., H.J.J., W.Q.P., H.Y.L., and H.D.C. supported the experiments of molecular and cellular biology. J.S.L. supported the animal experiments. Z‐R.L., B.S., H.T.T., and B.L.F. constructed the transgenic *T. gondii*. Y.X., Z‐R.L., and S.W. revised the manuscript. All authors read and approved the final manuscript.

## Supporting information

Supporting Information

Supporting Information

## Data Availability

All data needed to evaluate the conclusions in the paper are present in the paper and/or the Supplementary Materials.
