# Supplementary material for: The Toxoplasma Effector GRA4 Hijacks Host TBK1 to Oppositely Regulate Anti‐T. Gondii Immunity and Tumor Immunotherapy
Source: Adv Sci (Weinh). 2024 Jun 21;11(32):2400952. doi: 10.1002/advs.202400952 (PMC11348266; doi:10.1002/advs.202400952)
Supplement: Supplementary file 1 — Supporting Information [file ADVS-11-2400952-s002.docx]

**Supplementary Information**


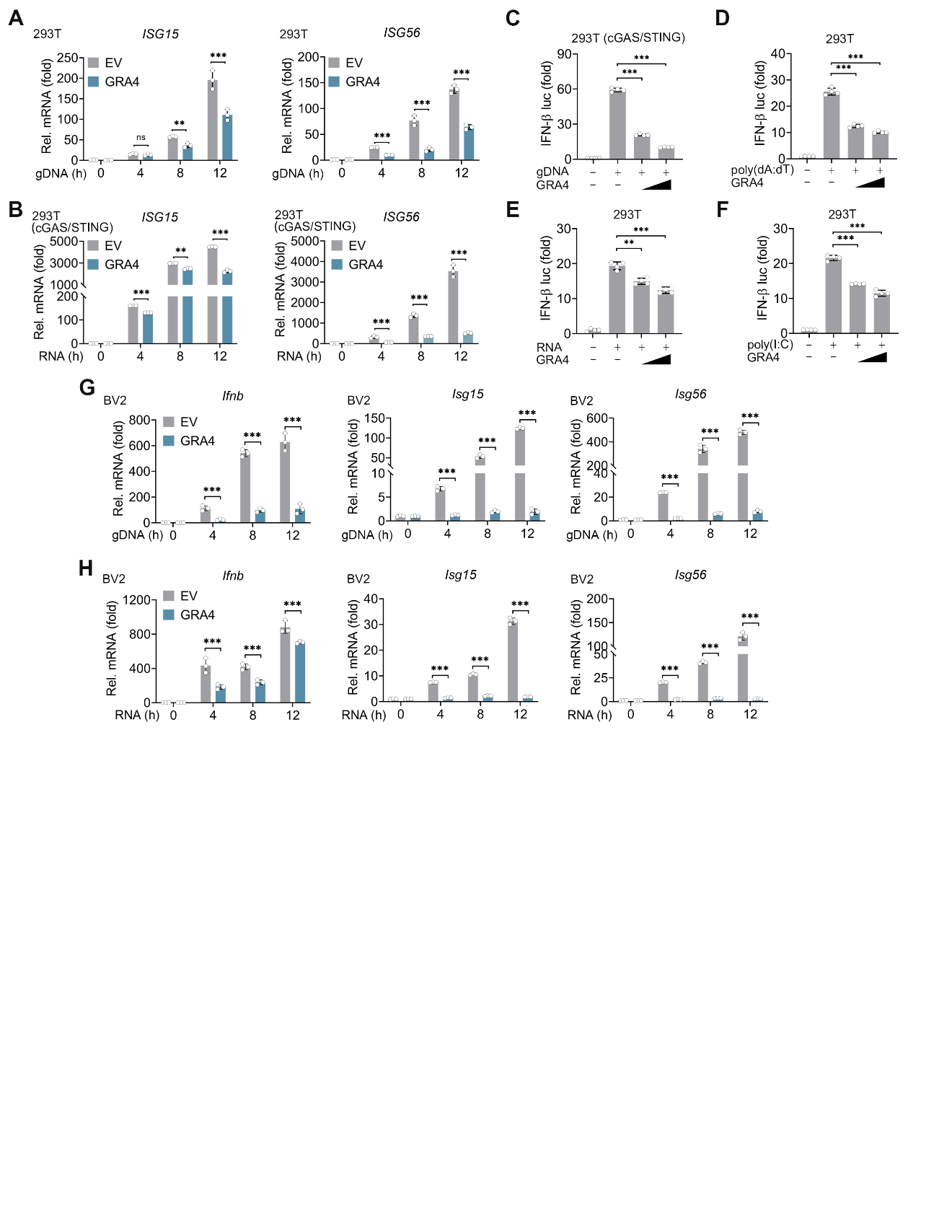


**Figure S1. GRA4 negatively regulates host IFN-I signaling. Related to Fig. 1.**

(A and B) qPCR analysis of *ISG15* and *ISG56* in 293T cells transfected with MYC-GRA4 or EV followed by ME49-gDNA (A) or -RNA (B) stimulation at indicated time points. (C) Luciferase activity in 293T cells (cGAS+STING stably expressing) transfected with a luciferase reporter for IFN-β luc, together with or without GRA4, followed by stimulation with or without ME49 gDNA for 8 h. Results are expressed relative to renilla luciferase activity. (D-F) Luciferase activity in 293T cells transfected with a luciferase reporter for IFN-β luc, together with or without GRA4, followed by treatment with or without poly(dA:dT) (D), ME49 RNA (E), or poly(I:C) (F) for 8 h. Results are expressed relative to renilla luciferase activity. (G and H) qPCR analysis of *Ifnb*, *Isg15*, and *Isg56* in BV2 cells transfected with MYC-GRA4 or EV followed by ME49 gDNA (G) and ME49 RNA (H) treatment at indicated time points. luc: luciferase. Data with error bars are represented as means ± SD. Each panel is a representative experiment of at least three independent biological replicates. ^**^*p* < 0.01, ^***^*p* < 0.001, and ns (not significant) as determined by unpaired Student’s t test.


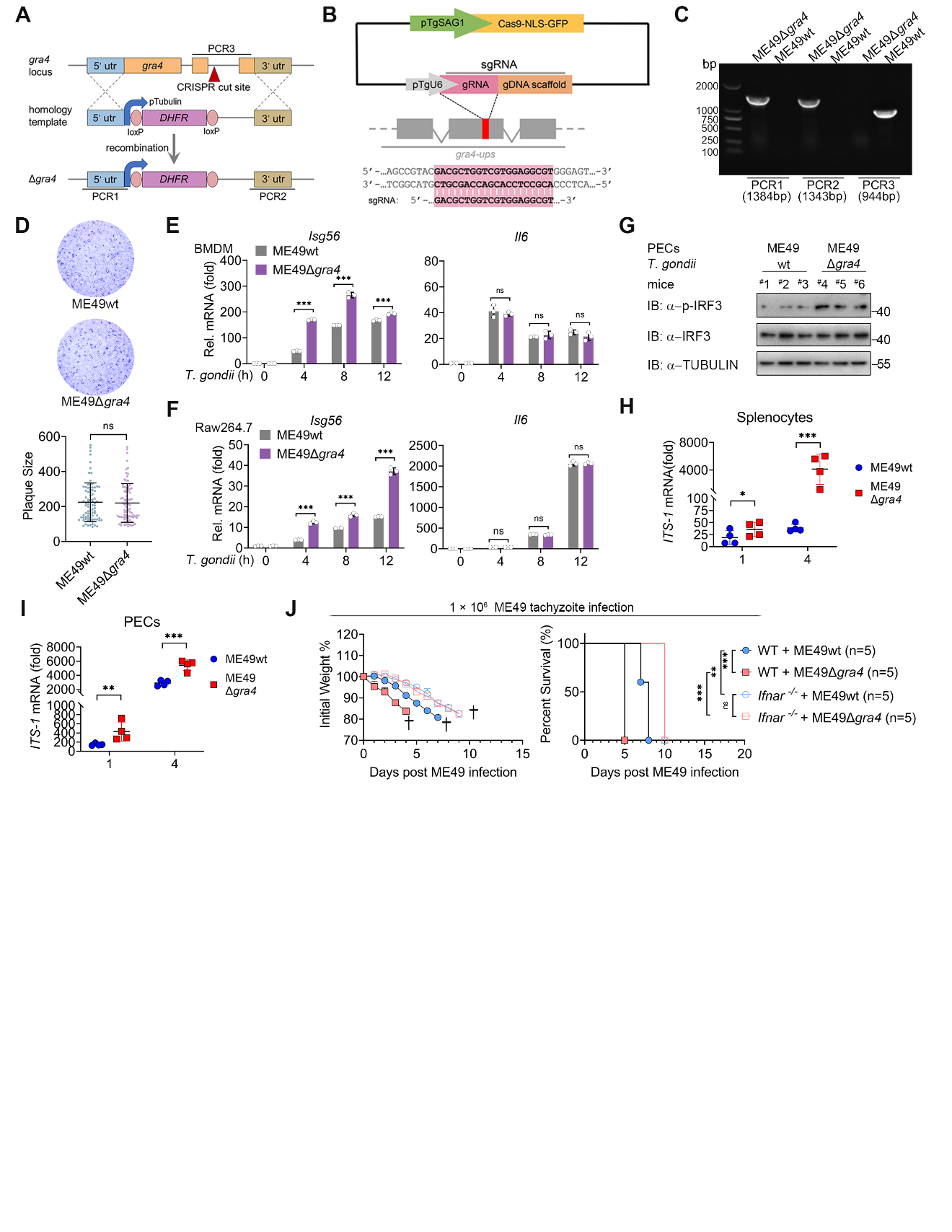


**Figure S2. GRA4 promotes anti-*T. gondii* immunity in an IFN-I dependent manner. Related to Fig. 1.**

(A) Schematic illustration of GRA4 depletion by CRISPR/Cas9‐mediated homologous gene replacement. (B) Schematic illustration of the plasmid expressing CAS9 and a single guide RNA (sgRNA) targeting the *gra4* gene in *T. gondii*. (C) Confirmation of gene knockout by diagnostic PCRs analysis of the ME49Δ*gra4* mutant. The parental strain ME49 was included as a control. (D) Plaque assay comparing the growth of ME49Δ*gra4* tachyzoites *in vitro* to that of the parental strain ME49wt. (E and F) qPCR analysis of *Isg56* and *Il6* in BMDMs (E) or Raw264.7 cells (F), followed by ME49wt or ME49*∆gra4* (MOI=5) infection at indicated time points. (G) Immunoblotting analysis of total and phosphorylated IRF3 in PECs from WT C57 mice infected with ME49wt or ME49*∆gra4*. ^#^1 to ^#^6 represents different mice, respectively. (H and I) Determination of *ITS-1* in splenocytes (H) and PECs (I) from WT C57 mice infected with ME49wt or ME49*∆gra4* on day 1 and day 4. (J) Body weights and survival rates of WT C57 (n=5) and *Ifnar^-/-^* C57 mice (n=5) infected with ME49wt or ME49∆*gra4* (1 × 10^6^) respectively as indicated, are shown. IB: immunoblotting. Data with error bars are represented as means ± SD. Each panel is a representative experiment of at least three independent biological replicates. ^*^*p* < 0.05, ^**^*p* < 0.01, ^***^*p* < 0.001, and ns (not significant) as determined by unpaired Student’s t test, two-way ANOVA, or the log rank test.


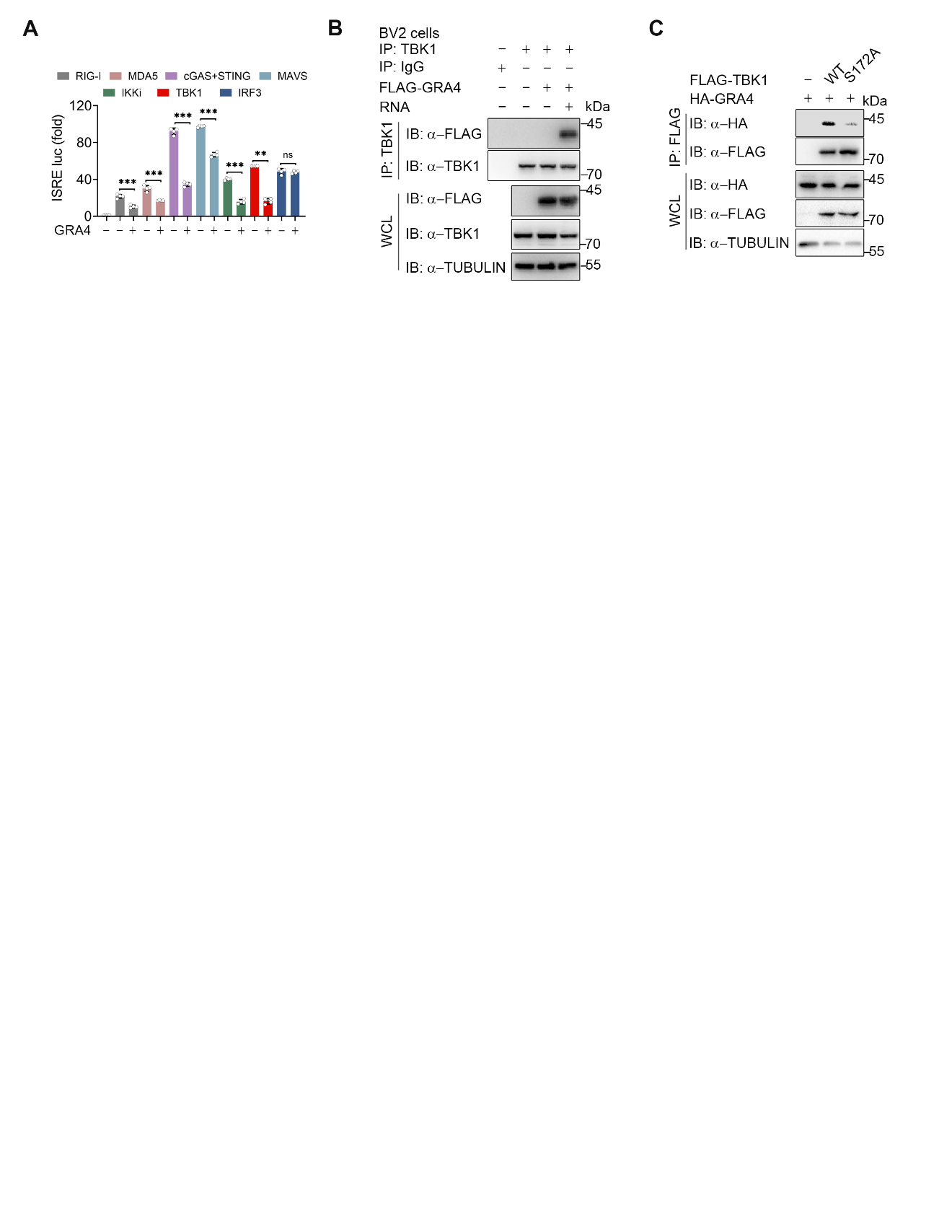


**Figure S3. GRA4 targets at activated TBK1. Related to Fig. 2.**

(A) Luciferase activity in 293T cells transfected with ISRE-luc, and FLAG-RIG-I, -MDA5, -MAVS, -cGAS plus STING, -TBK1, -IKKi, -IRF3 (5D), together with or without HA-GRA4. Results are expressed relative to renilla luciferase activity. (B) Immunoblotting analysis of BV2 cells transfected with FLAG-GRA4 or -EV, and treated with ME49 RNA for 8 h, followed by IP with anti-TBK1. (C) Immunoblotting analysis of 293T cells transfected with FLAG-WT or S172A mutant of TBK1, and HA-GRA4, followed by IP with anti-FLAG beads. luc: luciferase, IP: immunoprecipitation, WCL, whole cell lysis, IB: immunoblotting. Data with error bars are represented as means ± SD. Each panel is a representative experiment of at least three independent biological replicates. ^**^*p* < 0.05, ^***^*p* < 0.001 and ns (not significant) as determined by unpaired Student’s t test.


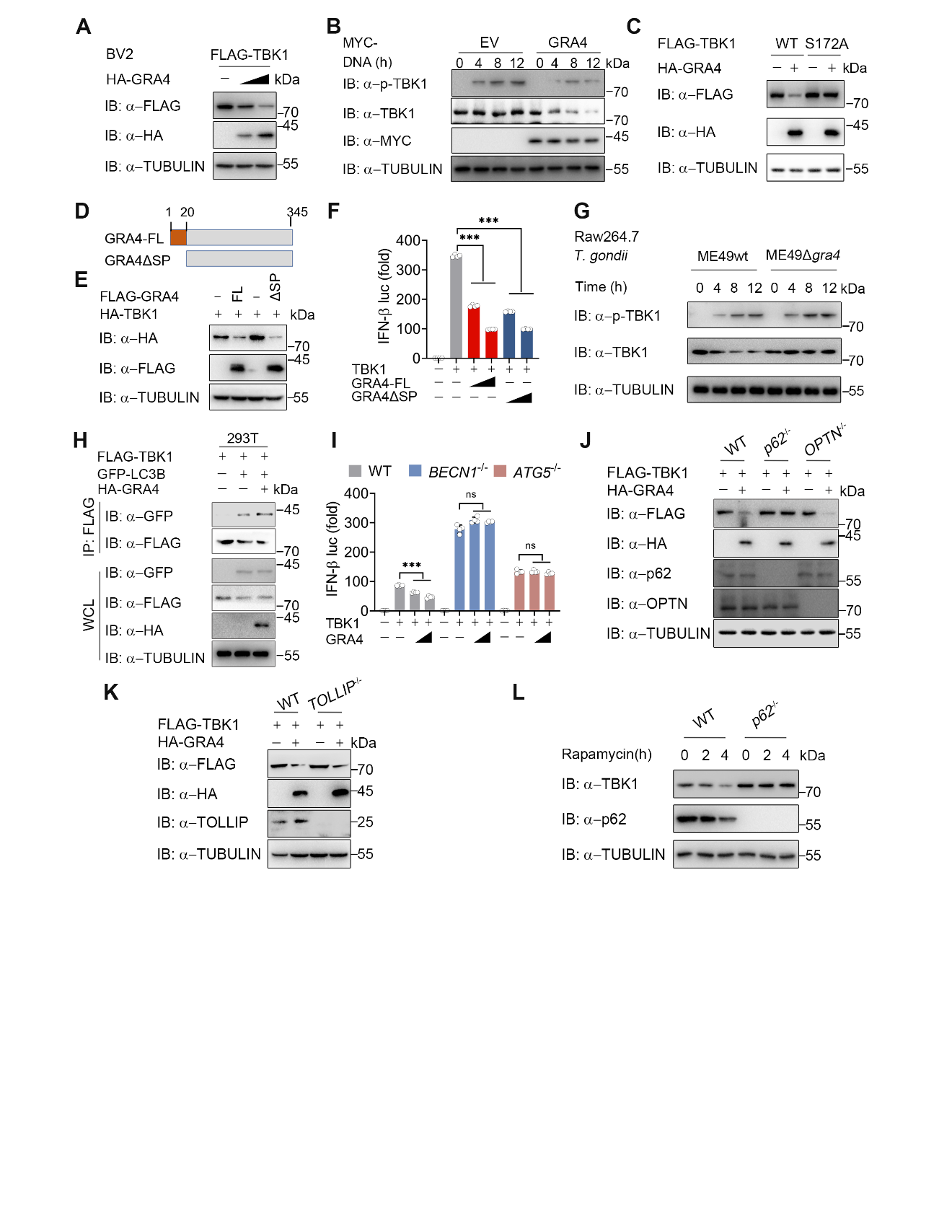


**Figure S4. Cargo receptor SQSTM1/p62 is indispensable for** **GRA4-induced autophagic degradation of TBK1. Related to Fig. 3.**

(A) Immunoblotting of TBK1 protein extracts of BV2 cells transfected with FLAG-TBK1, HA-EV or increasing amounts of HA-GRA4. (B) Immunoblotting analysis of total and phosphorylated TBK1 in 293T cells transfected with MYC-EV or -GRA4, followed by treatment with parasitic DNA at indicated time points. (C) Immunoblotting analysis of 293T cells transfected with FLAG-WT or -S172A mutant of TBK1, together with or without HA-GRA4. (D) The diagrammatic drawing of GRA4 deletion mutant. (E) Immunoblotting analysis of 293T cells transfected with WT or ∆SP mutant of FLAG-GRA4, and HA-TBK1. (F) Luciferase activity in 293T cells transfected with a luciferase reporter for IFN-β luc, HA- TBK1, together with FLAG-EV or increasing amounts of full length (FL) or ∆SP mutant FLAG-GRA4, is shown. (G) Immunoblotting analysis of total and phosphorylated TBK1 in Raw264.7 cells followed by ME49wt or ME49*∆gra4* (MOI=5) infection at indicated time points. (H) Immunoblotting analysis of 293T cells transfected with FLAG-TBK1, GFP-LC3B, and HA-GRA4, followed by IP with anti-FLAG beads. (I) Luciferase activity in WT, *BECN1* KO, or *ATG5* KO 293T cells transfected with a luciferase reporter for IFN-β luc, FLAG-TBK1, together with HA-EV or increasing amounts of HA-GRA4. (J and K) Immunoblotting analysis of WT, *p62* KO, *OPTN* KO (J) or *TOLLIP* KO (K) 293T cells transfected with FLAG-TBK1, together with HA-EV or HA-GRA4. (L) Immunoblotting analysis of WT and *p62* KO 293T cells, treated with rapamycin (250 nM) for indicated time points. luc: luciferase, IP: immunoprecipitation, WCL, whole cell lysis, IB: immunoblotting. Data with error bars are represented as means ± SD. Each panel is a representative experiment of at least three independent biological replicates. ^***^*p* < 0.001 and ns (not significant) as determined by unpaired Student’s t test.


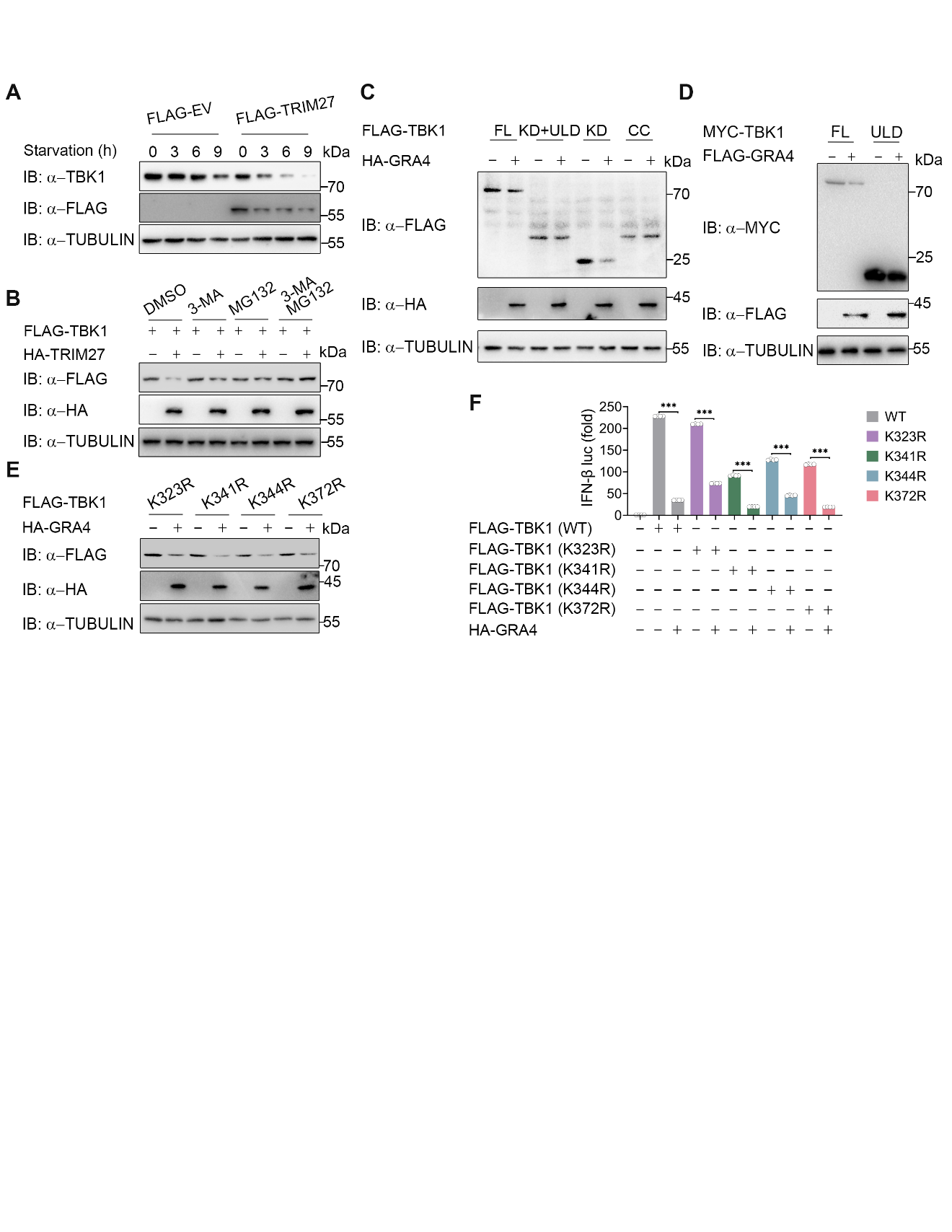


**Figure S5. TRIM27-catalyzed K48-ubiquitination at TBK1 Lys251/372 sites is prerequisite for GRA4 to degrade TBK1. Related to Fig. 4.**

(A) Immunoblotting analysis of total TBK1 in 293T cells transfected with FLAG-EV or TRIM27 for 24 h, starved for indicated time points with EBSS. (B) Immunoblotting analysis of 293T cells transfected with FLAG-TBK1, together with HA-EV or -TRIM27, followed by treatments of 3-MA (10 mM), bafilomycin A1 (Baf A1) (0.2 μM) and MG132 (10 μM), is shown. (C and D) Immunoblotting analysis of 293T cells transfected with various combinations of plasmid encoding FLAG or MYC-tagged Full Length (FL) or truncations of TBK1, HA- or FLAG-GRA4, is shown. (E) Immunoblotting analysis of 293T cells transfected with FLAG-K323R, -K341R, -K344R, and -K372R mutant of TBK1, together with HA-EV or -GRA4, is shown. (F) Luciferase activity in 293T cells transfected with a luciferase reporter for IFN-β-luc, FLAG-WT or -K323R, -K341R, -K344R and -K372R mutant of TBK1, together with HA-EV or -GRA4, is shown. luc: luciferase, IB: immunoblotting. Data with error bars are represented as means ± SD. Each panel is a representative experiment of at least three independent biological replicates. ^***^*p* < 0.001 as determined by unpaired Student’s t test.


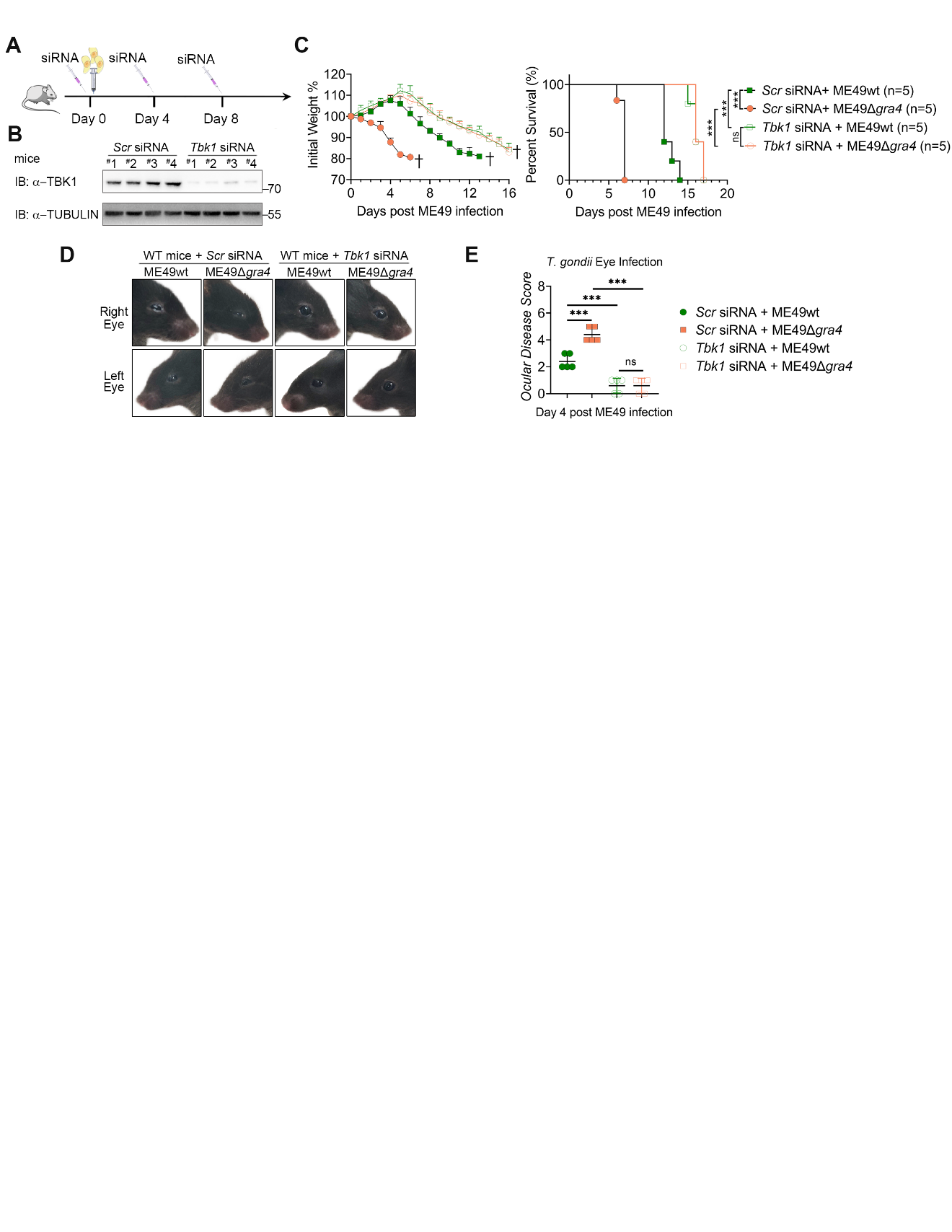


**Figure S6. The regulatory role of GRA4 in anti-*T. gondii* immunity relies on TBK1.**

(A) Schematic diagram showing the timing of *scramble* (*Scr*) or *Tbk1* small interfering RNA (siRNA) injections in the *T. gondii* infection model. Mice were intravenously injected with *Scr* or *Tbk1* siRNA (10 nM) at Day 0, 4, 8 post *T. gondii* infection. (B) The efficiency of *Tbk1* siRNAs for TBK1 in bone marrow cells was measured by immunoblotting. (C) The Body weights and survival rates of mice treated with *Scr* siRNA (n=5) and *Tbk1* siRNA (n=5) infected with ME49wt or ME49∆*gra4* (0.5×10^5^) respectively as indicated, are shown. (D and E) Representative micrographs of right and left eyes from mice at day 4 after treatment with *Scr* siRNA (n=5) and *Tbk1* siRNA (n=5) followed by ME49wt or ME49∆*gra4* (0.5×10^5^) infection (D), and the statistical analysis of ocular disease score (E), are as shown. IB: immunoblotting. Data with error bars are represented as means ± SD. Each panel is a representative experiment of at least three independent biological replicates. ^***^*p* < 0.001, and ns (not significant) as determined by unpaired Student’s t test, two-way ANOVA, or the log rank test.


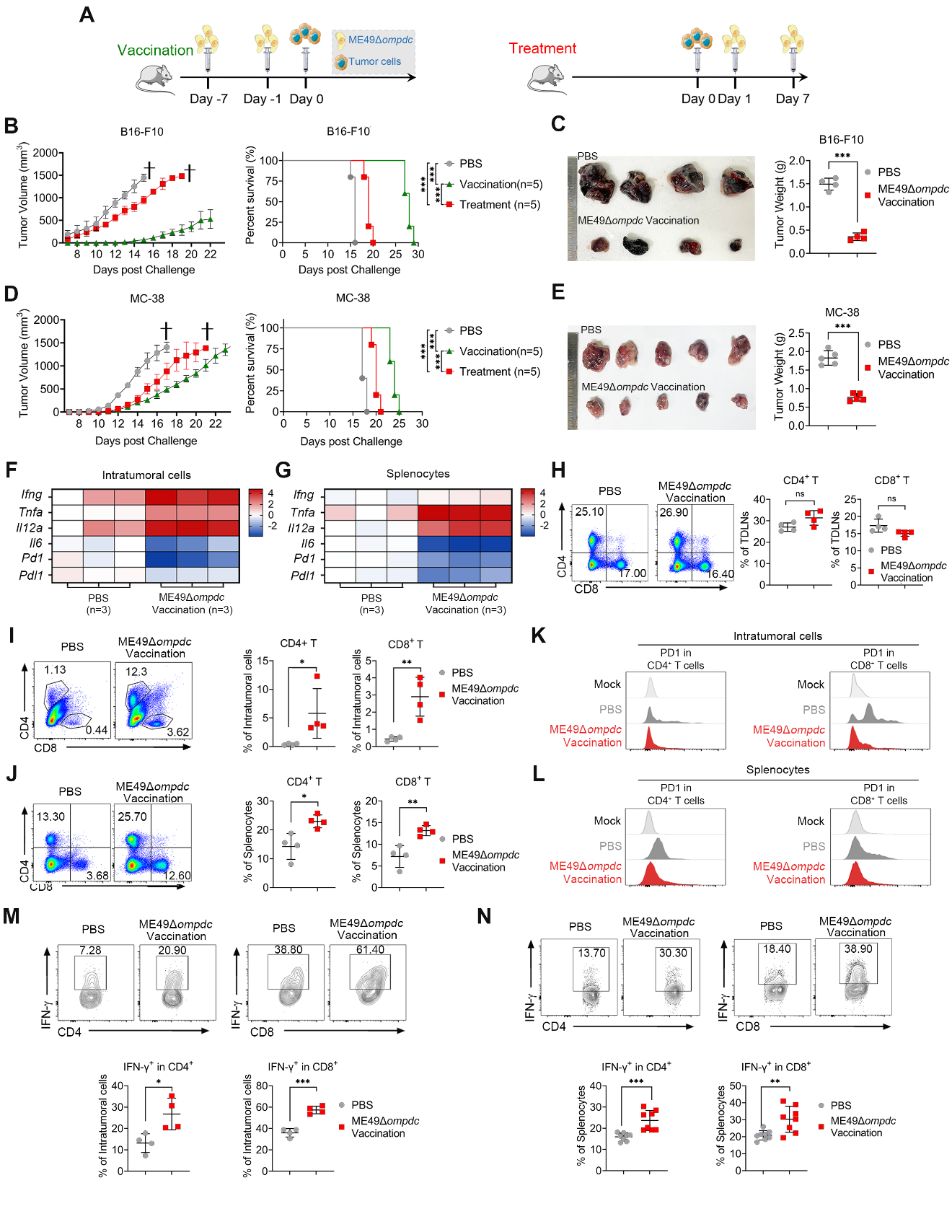


**Figure S7.** **Mice vaccinated with ME49Δ*ompdc* hold enhanced resistance to tumors in an IFN-I-dependent manner. Related to Fig. 5.** (A) Schematic of the “Vaccination” and “Treatment” schedules. Mice were treated with ME49Δ*ompdc* (2×10^6^ tachyzoites per mouse, *i.p.* injection) at day 1 and day 7 before (Vaccination group), or day 1 and day 7 post (Treatment group) inoculated subcutaneously with B16-F10 tumor cells (2×10^6^ tachyzoites per mouse, subcutaneously injection) on the right flank. (B) Tumor growth (left) and survival curves (right) of mice treated with ME49Δ*ompdc* or PBS *via* vaccination or treatment schedules, followed by implanted B16-F10 tumor cells. (C) The size (left) and weight analysis (right) of tumors dissected from mice treated with ME49Δ*ompdc* vaccination or PBS, followed by implanted B16-F10 tumor cells. (D) Tumor growth (left) and survival curves (right) of mice treated with ME49Δ*ompdc* or PBS *via* vaccination or treatment schedules, followed by implanted MC38 tumor cells. (E) The size (left) and weight analysis (right) of tumors dissected from mice treated with ME49Δ*ompdc* vaccination or PBS, followed by implanted MC38 tumor cells. (F and G) Heatmap showing qPCR results of indicated genes within intratumoral cells (F) or splenocytes (G) from mice injected with ME49Δ*ompdc* vaccination or PBS, followed by implanted B16-F10 tumor cells. (H-J)**,** Representative flow plots (left) and histogram (right) of CD4^+^ and CD8^+^ T cells in tumor-draining lymph nodes (TDLNs) (H) intratumoral cells (I) and splenocytes (J) from mice vaccinated with ME49Δ*ompdc* or PBS, followed by implanted B16-F10 tumor cells. (K and L) Representative quantification of PD-1 expression in CD4^+^ and CD8^+^ T cells in intratumoral cells (K) and splenocytes (L) from mice treated with ME49Δ*ompdc* vaccination or PBS, followed by implanted B16-F10 tumor cells. (M and N) Representative plots (up) and quantification (down) of IFN-γ of tumor-infiltrating CD4^+^ and CD8^+^ T cells in intratumoral cells (M) and splenocytes (N) from mice treated with ME49Δ*ompdc* vaccination or PBS, followed by implanted B16-F10 tumor cells. Data with error bars are represented as means ± SD. Each panel is a representative experiment of at least three independent biological replicates. ^*^*p* < 0.05, ^**^*p* < 0.01, ^***^*p* < 0.001, and ns (not significant) as determined by unpaired Student’s t test, two-way ANOVA, or by the log rank test.


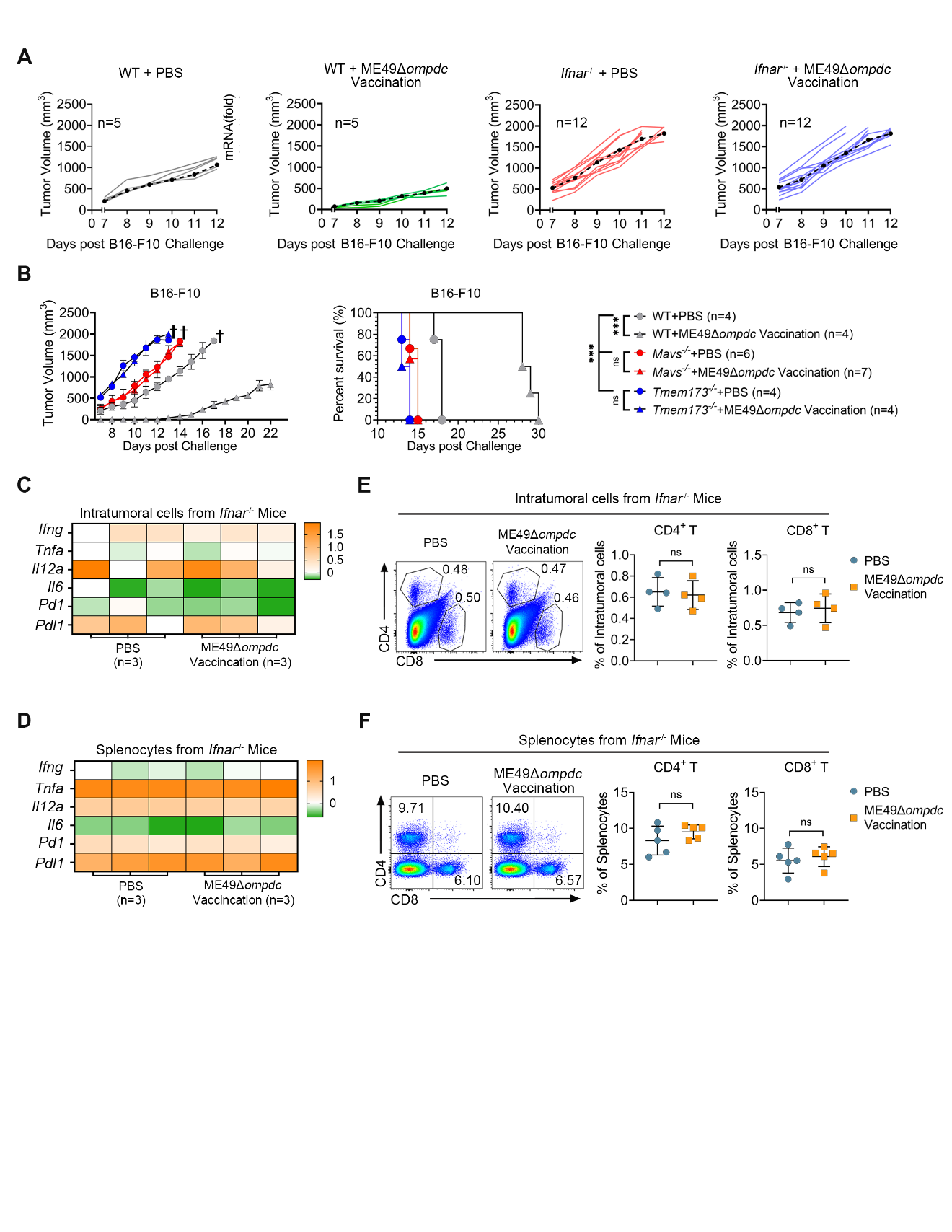


**Figure S8. Mice deficient in interferon α/β receptor (IFNAR) are insensitive to ME49Δ*ompdc* vaccination induced anti-tumor effect. Related to Fig. 5.** (A) Tumor growth of WT and *Ifnar*^-/-^ mice treated with ME49Δ*ompdc* vaccination or PBS, followed by implanted B16-F10 tumor cells. (B) Tumor growth (left) and survival curve (right) of WT, *Mavs*^-/-^ or *Tmem173*^-/-^ mice vaccinated with ME49Δ*ompdc* or PBS, followed by implanted B16-F10 tumor cells. (C and D) Heatmap showing qPCR results of indicated genes in intratumoral cells (C) and splenocytes (D) from *Ifnar*^-/-^ mice injected with ME49Δ*ompdc* vaccination or PBS, followed by implanted B16-F10 tumor cells. (E and F) Representative flow plots (left) and histogram (right) of CD4^+^ and CD8^+^ T cells in intratumoral cells (E) and splenocytes (F) from *Ifnar*^-/-^ mice vaccinated with ME49Δ*ompdc* or PBS, followed by implanted B16-F10 tumor cells. Data with error bars are represented as means ± SD. Each panel is a representative experiment of at least three independent biological replicates. ^***^*p* < 0.001, and ns (not significant) as determined by unpaired Student’s t test, two-way ANOVA, or by the log rank test.


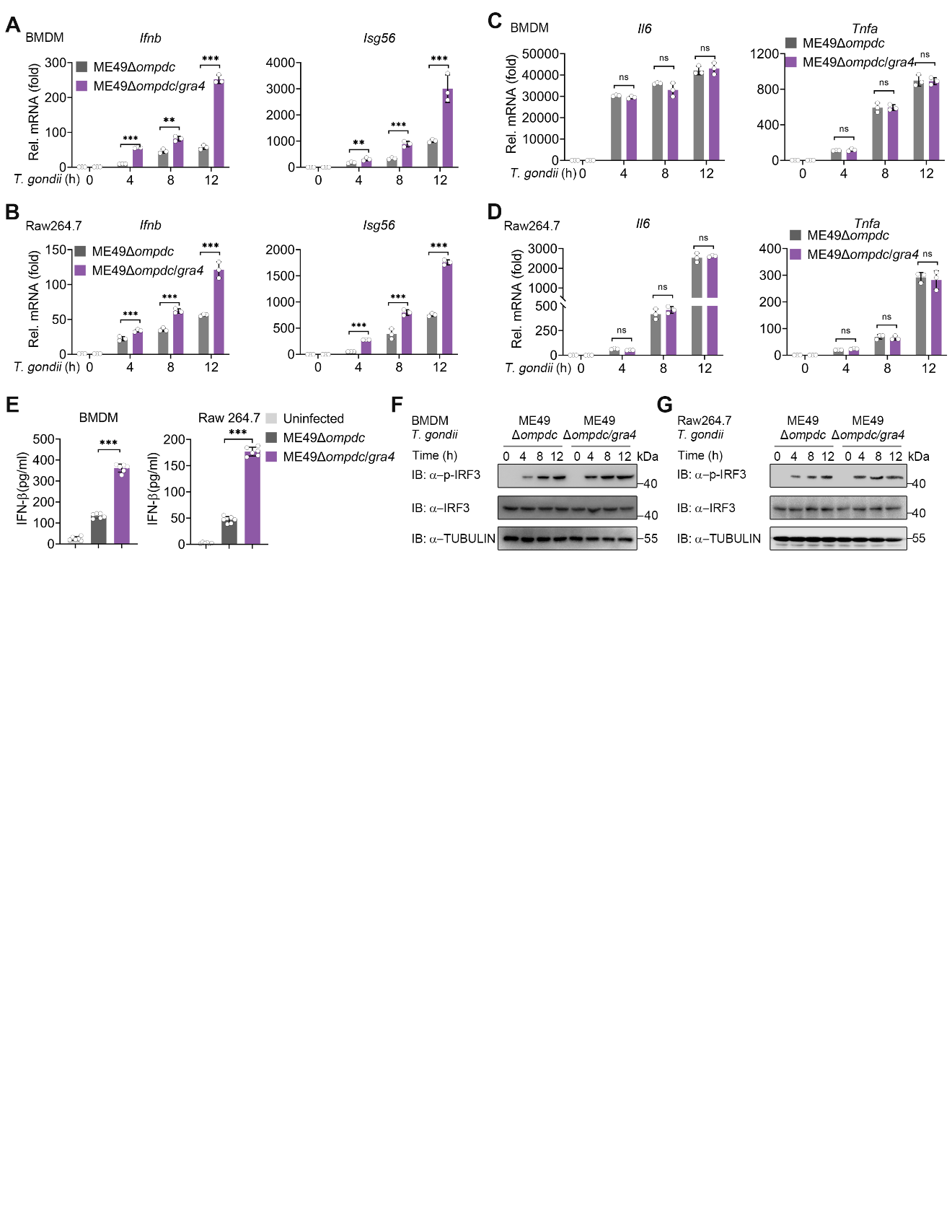


**Fig. S9.** **ME49Δ*ompdc*/*gra4* induced enhanced IFN-I responses within macrophages *in vitro*. Related to Fig. 5.** (A-D) qPCR analysis of *Ifnb*, *Isg56*, *Il6* and *Tnfa* in BMDMs (A and C) or Raw264.7 cells (B and D), followed by ME49Δ*ompdc* or ME49Δ*ompdc*/*gra4* (MOI=5) infection at indicated time points. (E) ELISA of IFN-β cytokines production in the supernatants of BMDMs or Raw264.7 cells, which were infected with ME49Δ*ompdc* or ME49Δ*ompdc*/*gra4* (MOI=5) for 24 h. (F and G) Immunoblotting analysis of total and phosphorylated IRF3 in BMDMs (F) or Raw264.7 cells (G) followed by ME49Δ*ompdc* or ME49Δ*ompdc*/*gra4* (MOI=5) infection at indicated time points. IB: immunoblotting. Data with error bars are represented as means ± SD. Each panel is a representative experiment of at least three independent biological replicates. ^**^*p* < 0.01, ^***^*p* < 0.001, and ns (not significant) as determined by unpaired Student’s t test.


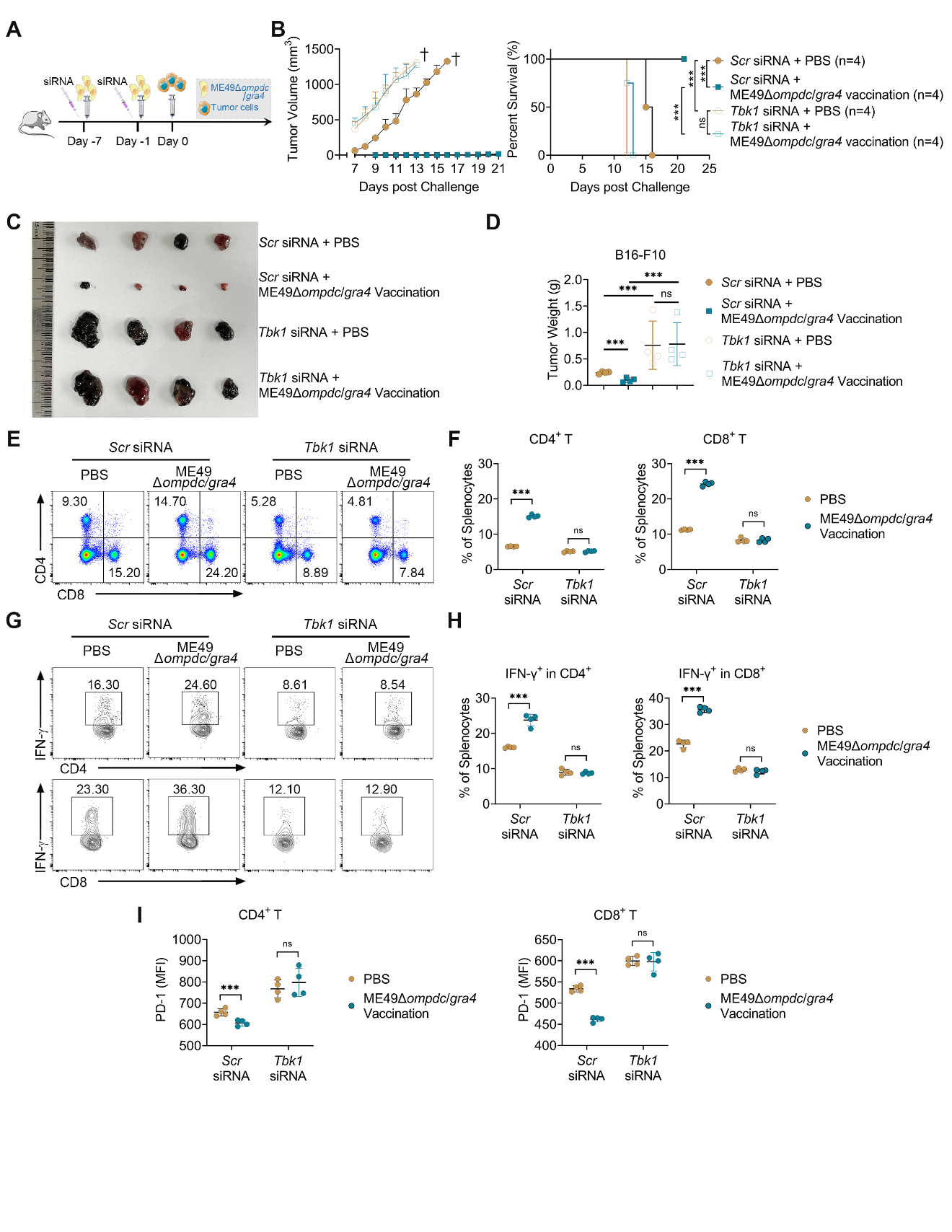


**Fig. S10. The anti-tumor effect of ME49Δ*ompdc*/*gra4* vaccination relies on TBK1.**

(A) Schematic diagram showing the timing of *Scr* or *Tbk1* small interfering RNA (siRNA) injections in the ME49Δ*ompdc*/*gra4* vaccination-induced anti-tumor model. Mice were intravenously injected with *Scr* or *Tbk1* siRNA (10 nM) at the same time with ME49Δ*ompdc*/*gra4* vaccination at Day -7, -1 before tumor-implanted. (B) Tumor growth (left) and survival curves (right) of mice pre-treated with *Scr* or *Tbk1* siRNA *via* ME49Δ*ompdc*/*gra4* vaccination, followed by implanted B16-F10 tumor cells. (C and D) The size (C) and weight analysis (D) of tumors dissected from mice pre-transfected with *Scr* or *Tbk1* siRNA *via* ME49Δ*ompdc*/*gra4* vaccination, followed by implanted B16-F10 tumor cells. (E and F) Representative flow plots (E) and histogram (F) of CD4^+^ and CD8^+^ T cells within splenocytes from mice vaccinated with pre-treated with *Scr* or *Tbk1* siRNA *via* ME49Δ*ompdc*/*gra4* vaccination, followed by implanted B16-F10 tumor cells. (G and H) Representative plots (G) and histogram (H) of IFN-γ of CD4^+^ T and CD8^+^ T cells within splenocytes from mice pre-treated with *Scr* or *Tbk1* siRNA *via* ME49Δ*ompdc*/*gra4* vaccination, followed by implanted B16-F10 tumor cells. (I) Quantification of PD-1 expression in CD4^+^ (left) and CD8^+^ (right) T cells from mice pre-treated with *Scr* or *Tbk1* siRNA *via* ME49Δ*ompdc*/*gra4* vaccination, followed by implanted B16-F10 tumor cells. Data with error bars are represented as means ± SD. Each panel is a representative experiment of at least three independent biological replicates. ^***^*p* < 0.001, and ns (not significant) as determined by unpaired Student’s t test, two-way ANOVA, or the log rank test.


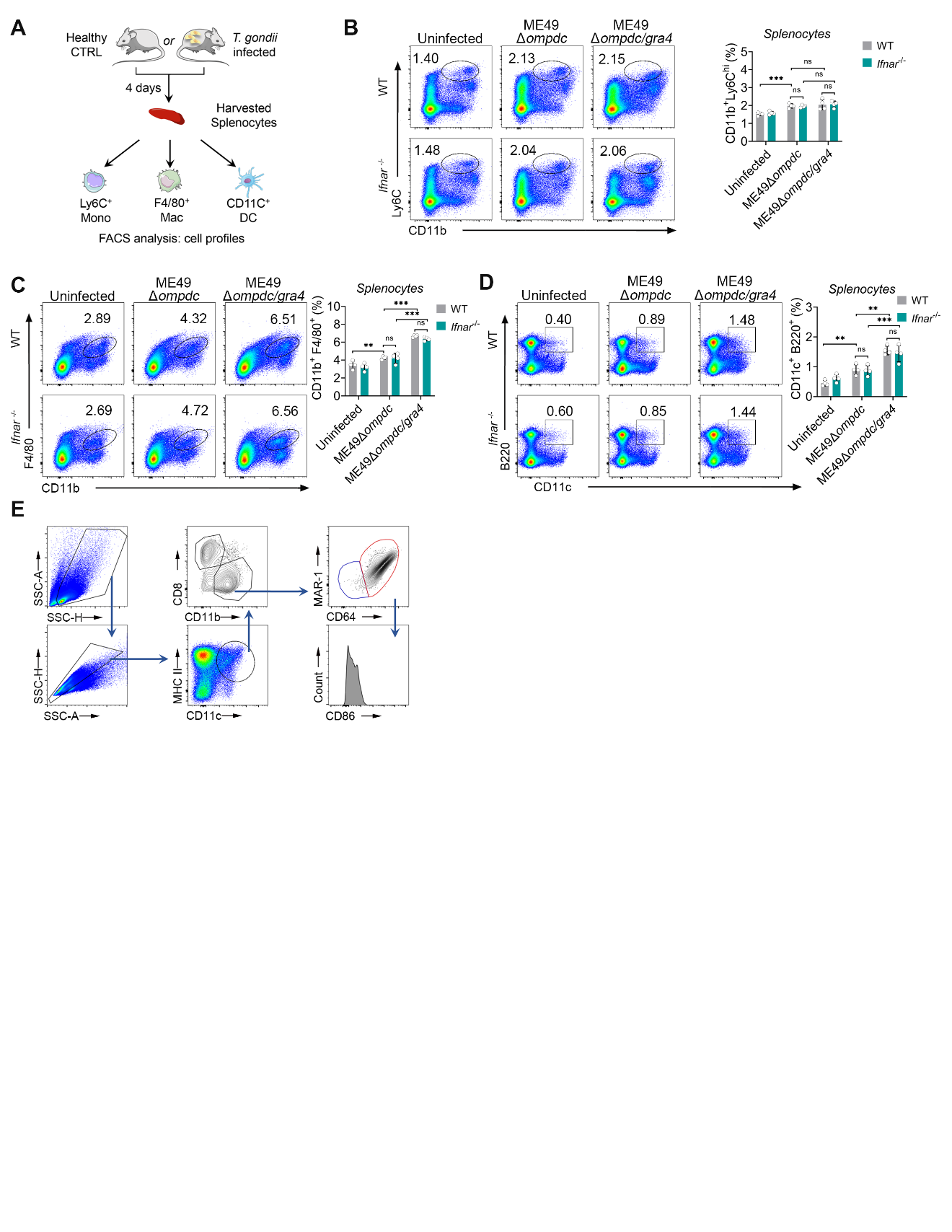


**Figure S11.** **Mice vaccinated with ME49Δ*ompdc*/*gra4* activate stronger IFN-I responses to promote proliferation of conventional dendritic cells (cDCs), but not monocytes, macrophages, and plasmacytoid dendritic cells (pDCs). Related to Fig. 6.** (A) Experimental model for FACS analysis of Ly6C^+^ monocytes, F4/80^+^ macrophages, and CD11C^+^ DCs sorted from the splenocytes and lymph nodes of mice with or without ME49Δ*ompdc/gra4* infection for 4 days. (B-D) Representative flow plots and histogram of monocytes (CD11b^+^Ly6C^hi^) (B), macrophages (CD11b^+^F4/80^+^) (C), and pDCs (CD11c^+^B220^+^) (D) in splenocytes from WT or *Ifnar*^-/-^ mice with or without ME49Δ*ompdc* or ME49Δ*ompdc*/*gra4* infection. (E) Representative flow cytometry gating strategy for CD64^+^MAR-1^+^CD11b^+^ DCs. Data with error bars are represented as means ± SD. Each panel is a representative experiment of at least three independent biological replicates. ^**^*p* < 0.01, ^***^*p* < 0.001, and ns (not significant) as determined by unpaired Student’s t test.


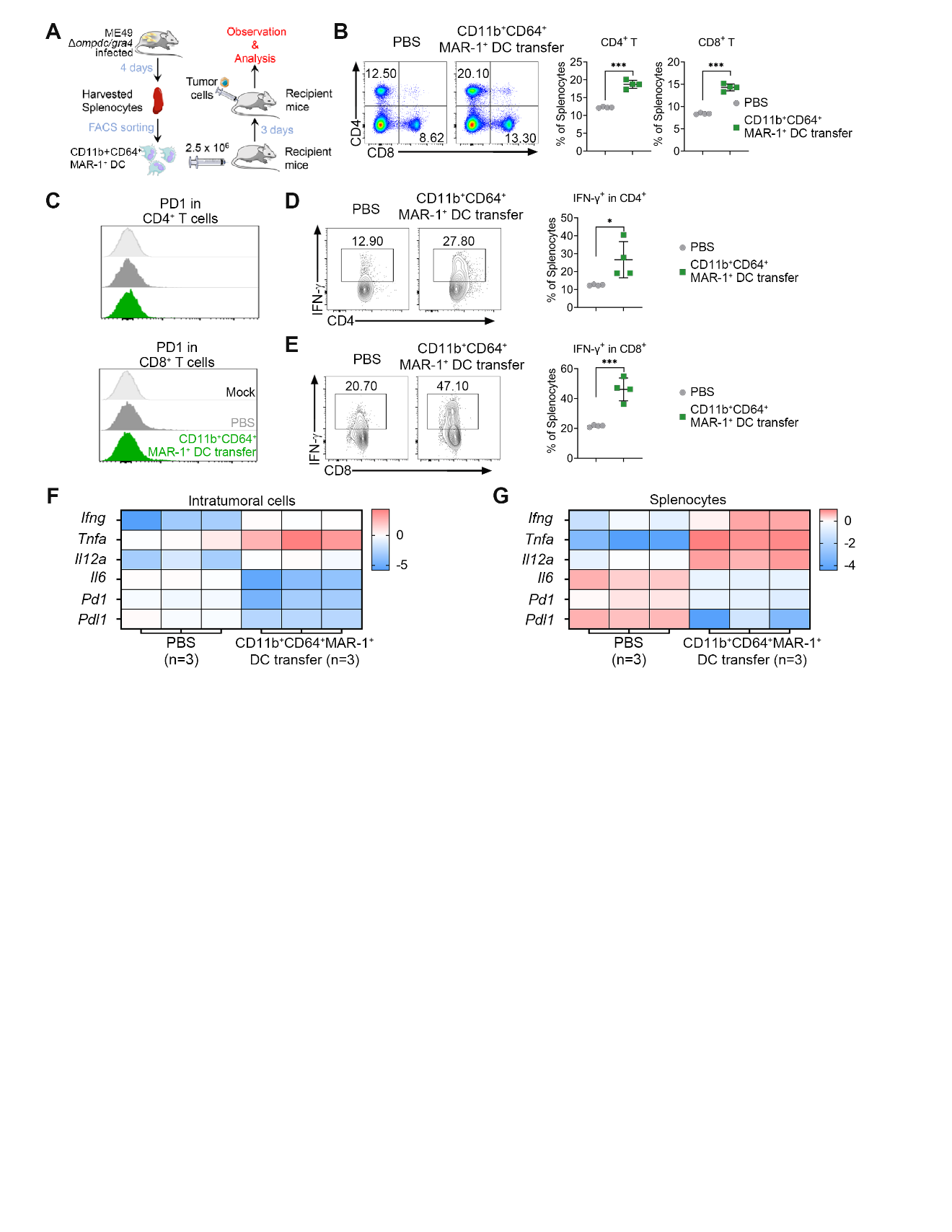


**Figure S12. CD64^+^MAR-1^+^CD11b^+^ DCs induced by ME49Δ*ompdc*/*gra4* vaccination trigger efficient anti-tumor immunity. Related to Fig. 6.** (A) Experimental design for adoptive transfer in mice. (B) Representative flow plot (left) and histogram (right) of CD4^+^ and CD8^+^ T cells in splenocytes from recipient mice with or without CD64^+^MAR-1^+^CD11b^+^ DCs adoptive transfer, followed by implanted B16-F10 tumor cells. (C) Representative flow cytometric analysis of PD-1 in CD4^+^ (up) and CD8^+^ (down) T cells in splenocytes from recipient mice with or without CD64^+^MAR-1^+^CD11b^+^ DCs adoptive transfer, followed by implanted B16-F10 tumor cells. (D and E) Representative flow plots and histogram of IFN-γ of CD4^+^ T (D) and CD8^+^ T (E) cells in splenocytes from recipient mice with or without CD64^+^MAR-1^+^CD11b^+^ DCs adoptive transfer, followed by implanted B16-F10 tumor cells. (F and G) Heatmap showing qPCR results of indicated genes in intratumoral cells (F) and splenocytes (G) from recipient mice with or without CD64^+^MAR-1^+^CD11b^+^ DCs adoptive transfer, followed by implanted B16-F10 tumor cells. Data with error bars are represented as means ± SD. Each panel is a representative experiment of at least three independent biological replicates. ^*^*p* < 0.05, ^***^*p* < 0.001, as determined by unpaired Student’s t test.
