# Supplementary material for: The Toxoplasma Effector GRA4 Hijacks Host TBK1 to Oppositely Regulate Anti‐T. Gondii Immunity and Tumor Immunotherapy
Source: Adv Sci (Weinh). 2024 Jun 21;11(32):2400952. doi: 10.1002/advs.202400952 (PMC11348266; doi:10.1002/advs.202400952)
Supplement: Supplementary file 2 — Supporting Information [file ADVS-11-2400952-s001.docx]

**Tables:**

**Table 1. Plasmids for ME49 mutants construction.**

| **Name of plasmids** | **Use** | **Construction methods** |
| --- | --- | --- |
| pSAG1-Cas9-sgMIC3 | Template for gene-specific CRISPR plasmid construction | Shen et al. ^67^ |
| pSAG1-Cas9-sgGRA4 | *GRA4* specific CRISPR plasmid for GRA4 knockout | Site-directed mutagenesis to replace the gRNA in pSAG1-Cas9-sgMIC3 with gene specific gRNAs. |
| pUC19 | Template for pUC19 amplification | From the Sibley Lab |
| pGRA4::*DHFR* | Homologous template for *GRA4* replacement by *DHFR* | PCR derived fragments 5H GRA4, DHFR, 3H GRA4 were cloned into pUC19 |
| pLinker-AID-3xHA-DHFR-LoxP | Template for Loxp-DHFR-Loxp amplification | From the Sibley Lab |

**Table 2. Targeting sequences of shRNA- or sgRNA- used in this study.**

| shRNA-targeting gene | Sequences (5’ → 3’) |
| --- | --- |
| *Scr* | AACAAGATGAAGAGCACCAAC |
| *TRIM27* | AGGGCTGAAAGAATCAGGATC |
| *TRIP* | CCAGCATGGTTACTACGAAAC |
| *RNF41* | CCTGGAGGAGACAATTGAATA |
| *RNF128* | TCTTAACGTGCAACCATATTT |
| *NEDD4* | TGCAAGCACAACGTGCATTTA |
| *TRIM23* | GCAGTCATAGAGACAGAATTA |
| sgRNA-targeting gene | Sequences (5’ → 3’) |
| *OPTN* | CACCGAAACCTGGACACGTTTACCC |
| *TOLLIP* | CACCGACCACCGTCAGCACTCAGCG |
| *TBK1* | CACCGCATAAGCTTCCTTCGTCCAG |

**Table 3. Sequences of qRT-PCR primers used in this study.**

| **Primer sequences for qRT-PCR in human** | |
| --- | --- |
| **Name** | **Sequences (5’** **→ 3’)** |
| *IFNB*-F | GCTTGGATTCCTACAAAGAAGCA |
| *IFNB*-R | ATAGATGGTCAATGCGGCGTC |
| *ISG15*-F | CGCAGATCACCCAGAAGATCG |
| *ISG15*-R | TTCGTCGCATTTGTCCACCA |
| *ISG56*-F | TTGATGACGATGAAATGCCTGA |
| *ISG56*-R | CAGGTCACCAGACTCCTCAC |
| *GAPDH*-F | GGAGCGAGATCCCTCCAAAAT |
| *GAPDH*-R | GGCTGTTGTCATACTTCTCATGG |
| **Primer sequences for qRT-PCR in mouse** | |
| **Name** | **Sequences (5’** **→ 3’)** |
| *Ifnb*-F | TCACCTACAGGGCGGACTTC |
| *Ifnb*-R | GGTGTCCGTGACTAACTCCAT |
| *Isg15*-F | GGTGTCCGTGACTAACTCCAT |
| *Isg15*-R | CTGTACCACTAGCATCACTGTG |
| *Isg56-*F | TGCGATCCACAGTGAACAAC |
| *Isg56-*R | ACTTCCGGGAAATCGATGAG |
| *Ifna2*-F | CTTACTCAGCAGACCTTGAACC |
| *Ifna2*-R | CTGCTGCATCAGACAGGTTT |
| *Il6*-F | CCAGTTTGGTAGCATCCATC |
| *Il6*-R | CTCTGGGAAATCGTGGAAAT |
| *Tnfa*-F | GACGTGGAACTGGCAGAAGAG |
| *Tnfa*-R | TTGGTGGTTTGTGAGTGTGAG |
| *Pd1*-F | ACCCTGGTCATTCACTTGGG |
| *Pd1*-R | CATTTGCTCCCTCTGACACTG |
| *Pdl1*-F | GCTCCAAAGGACTTGTACGTG |
| *Pdl1*-R | TGATCTGAAGGGCAGCATTTC |
| *Ifng*-F | ATGAACGCTACACACTGCATC |
| *Ifng*-R | CCATCCTTTTGCCAGTTCCTC |
| *Il12a*-F | CATCGATGAGCTGATGCAGT |
| *Il12a*-F | CAGATAGCCCATCACCCTGT |
| *Gapdh*-F | AAGGTCATCCCAGAGCTGAA |
| *Gapdh*-R | CTGCTTCACCACCTTCTTGA |
| **qRT-PCR Primer sequences for *T. gondii* detection** | |
| **Name** | **Sequences (5’** **→ 3’)** |
| *ITS-1*-F | AATATTGGAAGCCAGTGCAGG |
| *ITS-1*-R | CAATCTTTCACTCTCTCTCAA |

**Table 4. Antibodies for immunoprecipitation, immunoblot and immunofluorescence assays.**

| Antibodies | Supplier | Cat. & RRID |
| --- | --- | --- |
| Rabbit monoclonal anti-TBK1/NAK (phospho S172) | Cell Signaling Technology | Cat#5483; RRID: AB_10693472 |
| Rabbit monoclonal anti-TBK1/NAK | Cell Signaling Technology | Cat#3013; RRID: AB_2199749 |
| Rabbit monoclonal anti-IRF3 (phospho S396) | Cell Signaling Technology | Cat# 4947; RRID: AB_823547 |
| Rabbit monoclonal anti-IRF3 | Cell Signaling Technology | Cat# 11904; RRID: AB_2722521 |
| Mouse monoclonal anti-Flag (M2) peroxidase (HRP) | Sigma-Aldrich | Cat# 5483P; RRID: AB_10693472 |
| Hemagglutinin (HA)-HRP antibody | Roche Applied Science | Cat# 12994; RRID: AB_2630393 |
| Mouse monoclonal anti-Myc Tag | Cell Signaling Technology | Cat#3739; RRID: AB_10889248 |
| Mouse monoclonal anti-p62 | Cell Signaling Technology | Cat# 88588; RRID: AB_2800125) |
| Rabbit polyclonal anti- TBK1 | Proteintech | Cat# 67211-1-Ig; RRID: AB_2882504 |
| Rabbit polyclonal anti- NDP52 | Proteintech | Cat# 12229-1-AP; RRID: AB_11182600 |
| Rabbit polyclonal anti-OPTN | Proteintech | Cat# 10837-1-AP; RRID: AB_2156665 |
| Rabbit polyclonal anti-TOLLIP | Proteintech | Cat# 11315-1-AP; RRID: AB_2256373 |
| Rabbit polyclonal anti-ATG5 | Proteintech | Cat# 10181-2-AP; RRID: AB_2062045 |
| Rabbit polyclonal anti-BECLIN1 | Proteintech | Cat# 11306-1-AP; RRID: AB_2259061 |
| Mouse monoclonal anti-Ubiquitin | Cell Signaling Technology | Cat# 3936; RRID: AB_331292 |
| Mouse monoclonal anti-K48- Ubiquitin | Cell Signaling Technology | Cat# 8081; RRID: AB_10859893 |
| Mouse monoclonal anti-TUBULIN | Santa Cruz Biotechnology | Cat# sc-5274; RRID: AB_2288090 |
| Goat anti-rabbit | Abcam | Cat# ab6721; RRID: AB_955447 |
| Goat anti-mouse | Abcam | Cat# ab6789; RRID: AB_955439 |
| Goat anti-mouse IgG H&L (Alexa Fluor® 594) | Abcam | Cat# ab150116; RRID: AB_2650601 |
| Goat anti-rat IgG H&L (Alexa Fluor® 488) | Abcam | Cat# ab150165; RRID: AB_2650997 |
| Goat anti-rabbit IgG H&L (Alexa Fluor® 547) | Abcam | Cat# ab150079; RRID: AB_2722623 |

**Table 5. Antibodies for flow cytometry and cell sorting.**

| Antibodies | Supplier | Cat. & RRID |
| --- | --- | --- |
| Anti-mouse CD8a Monoclonal Antibody (53-6.7), APC | eBioscience | Cat# 17-0081-82; RRID: AB_469335 |
| Anti-mouse CD4  CD4 Monoclonal Antibody (RM4-5), eFluor™ 450 | eBioscience | Cat# 48-0042-82; RRID: AB_1272194 |
| Anti-mouse IFN gamma Monoclonal Antibody (XMG1.2), PE | eBioscience | Cat# 12-7311-82; RRID: AB_466193 |
| Anti-mouse CD279 (PD-1) Monoclonal Antibody (RMP1-30), PE | eBioscience | Cat# 12-9981-82; RRID: AB_466290 |
| Anti-mouse CD11b Monoclonal Antibody (M1/70), eFluor™ 450 | eBioscience | Cat# 48-0112-82; RRID: AB_1582236 |
| Anti-mouse Ly-6C Monoclonal Antibody (HK1.4), PerCP-Cyanine5.5 | eBioscience | Cat# 45-5932-82; RRID: AB_2723343 |
| Anti-mouse F4/80 Monoclonal Antibody (BM8), FITC, | eBioscience | Cat# 11-4801-82; RRID: AB_2637191 |
| Anti-mouse CD45R (B220) Monoclonal Antibody (RA3-6B2), eFluor™ 450 | eBioscience | Cat# 48-0452-82; RRID: AB_1548761 |
| Anti-mouse CD11c Monoclonal Antibody (N418), APC | eBioscience | Cat# 17-0114-82; RRID: AB_469346 |
| Anti-mouse MHC Class II (I-A/I-E) Monoclonal Antibody (M5/114.15.2), PE | eBioscience | Cat# 12-5321-81, RRID: AB_465927 |
| Anti-mouse FcεRIα Monoclonal Antibody (MAR-1), APC-Cy7 | BioLegend | Cat# 134325; RRID: AB_2572063 |
| Anti-mouse CD86 Monoclonal Antibody (GL-1), FITC | BioLegend | Cat# 105006; RRID: AB_313149 |
